# A new meta-module design for efficient reconfiguration of modular robots

**DOI:** 10.1007/s10514-021-09977-6

**Published:** 2021-03-22

**Authors:** Irene Parada, Vera Sacristán, Rodrigo I. Silveira

**Affiliations:** 1 Department of Mathematics and Computer Science, TU Eindhoven, Eindhoven, The Netherlands; 2 Departament de Matemàtiques, Universitat Politècnica de Catalunya, Barcelona, Spain

**Keywords:** Geometric reconfiguration, Self-reconfiguring modular robots, Meta-modules

## Abstract

We propose a new meta-module design for two important classes of modular robots. The new meta-modules are three-dimensional, robust and compact, improving on the previously proposed ones. One of them applies to so-called *edge-hinged* modular robot units, such as M-TRAN, SuperBot, SMORES, UBot, PolyBot and CKBot, while the other one applies to so-called *central-point-hinged* modular robot units, which include Molecubes and Roombots. The new meta-modules use the rotational degrees of freedom of these two types of robot units in order to expand and contract, as to double or halve their length in each of the two directions of its three dimensions, therefore simulating the capabilities of Crystalline and Telecube robots. Furthermore, in the edge-hinged case we prove that the novel meta-module can also perform the scrunch, relax and transfer moves that are necessary in any tunneling-based reconfiguration algorithm for expanding/contracting modular robots such as Crystalline and Telecube. This implies that the use of meta-meta-modules is unnecessary, and that currently existing efficient reconfiguration algorithms can be applied to a much larger set of modular robots than initially intended. We also prove that the size of the new meta-modules is optimal and cannot be further reduced.

**Supplementary Information:**

The online version supplementary material available at 10.1007/s10514-021-09977-6.

## Introduction

Self-reconfigurable modular robots are sets of robotic units attached to each other forming a connected shape called robot *configuration*. The units can change their connectivity, moving relative to each other, thus changing the shape of the robot. By modifying their morphology (reconfiguring) they can better suit different tasks, adapt to different environments, and self-repair. This makes them more versatile than fixed-shape unique-purpose robots (Murata and Kurokawa 2012; Yim et al. 2007).

**Fig. 1:**
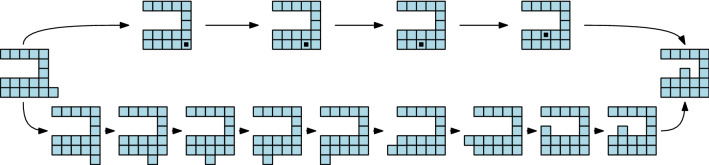
Different reconfiguration strategies. Top: tunneling algorithm. The black dot represents that two units are compressed in the same cell. Bottom: the same reconfiguration achieved by a surface algorithm

Self-reconfigurable modular robots have been classified according to different criteria: their architecture and topology, the nature of their connections, their degrees of freedom, their propulsion methods, etc. (Brunete et al. 2017; Mounarak and Ben-Tzvi 2012; Murata and Kurokawa 2012; Sirajoulis and Adamatzky 2015). From a geometric viewpoint, a very interesting class of modular robots is that of those that are able to expand and contract, since this property can be exploited by reconfiguration algorithms. The capability to expand and contract allows moving the units through the interior of the robot configuration, as shown in the top row of Fig. 1. This allows so-called *tunneling* reconfiguration algorithms, where modules travel through the volume of the robot. The bottom row of the figure shows how the same reconfiguration is achieved by means of traversing the surface of the robot, i.e., by moving the units along the boundary of the configuration, requiring a longer sequence of steps.

This tunneling capability is particularly interesting because it can be exploited to reconfigure robotic systems *in-place*, i.e., using only the space occupied by the initial and goal configurations, with a small number of robot moves and parallel steps. As the volume of a robot configuration grows, the surface area per module becomes proportionally smaller. This is a limitation to parallelism in surface reconfiguration strategies that causes the reconfiguration speed to decrease as the number of modules increases.

Instances of self-reconfiguring systems with square or cubic units that can expand and contract by a factor of two in each of its dimensions are Crystalline robots (Rus and Vona 2001) in two dimensions and Telecubes (Suh et al. 2002) in three dimensions. Other modular robots that can expand and contract include the Metamorphic robot (Pamecha et al. 1996), which can squeeze by modifying the angles of its 2-dimensional hexagonal pieces; PolyPod (Yim 1994), a chain bipartite robot made of contractible segments with two degrees of freedom; TETROBOT (Hamlin and Sanderson 1998), a truss-structured robot composed of rigid or actuated struts and joints allowing rotation; Odin (Lyder et al. 2008), a versatile three-dimensional robot using telescopic links; and the very recent M-Lattice (Yang et al. 2018), a 2-dimensional modular robot made of triangular units that can modify its angles as well as contract and expand its edges.

Several tunneling algorithms for reconfiguring Crystalline and Telecube robots have been proposed in the past. In all of them the units are grouped into so-called *meta-modules* (Kotay and Rus 2000; Nguyen et al. 2000). These are connected configurations of at least $2\times 2$ equal units in the 2D case, and $2\times 2 \times 2$ units in 3D, that collaborate in order to form a robot that has more functionality (e.g., in terms of possible moves) than any single module (Nguyen et al. 2000), adding extra versatility to the robotic system.

Many reconfiguration strategies have been proposed in the literature. The *melt-grow* method (Rus and Vona 2001) is a centralized algorithm that allows to reconfigure any connected robot with *n* units in $O(n^2)$ moves and steps. The *Pac-Man* algorithm (Butler and Rus 2003) and the algorithm by Vassilvitskii et al. (2002) both also reconfigure in $O(n^2)$ parallel steps. In-place reconfiguration is also possible. Assuming constant velocity and strength, under which a module can pull or push only a constant number of other modules at constant speed, meta-modules made of $2\times 2 (\times 2)$ units can be used to reconfigure in-place. This can be achieved by both a centralized (Aloupis et al. 2011) and a distributed (Perera 2015) algorithm. The overall number of moves performed by these algorithms is $\varTheta (n^2)$, which is optimal in this setting. If the modules of the robot have linear strength, the total number of moves can be reduced to *O*(*n*) (Aloupis et al. 2009). If, in addition, we allow velocities to build up over time, then reconfiguration is possible in $O(\log n)$ parallel steps and $O(n \log n)$ overall moves (Aloupis et al. 2008).

Many current modular robot prototypes have other very convenient properties such as chain/tree or hybrid architectures providing their units with high mobility or locomotion capabilities, but they cannot expand and contract. However, this can be achieved by combining them into meta-modules.

In general, the meta-module design problem aims at structuring a set of modular robot units in a way such that this structured set is able to perform the operations required in reconfiguration algorithms. In our case, the goal is to design minimum-size meta-modules that can perform the Crystalline and Telecube unit operations *expand*, *contract*, *attach* and *detach* and that are valid for a wide range of modular robots. This will allow to apply the previously described algorithms, designed for robots that can expand and contract, also to other important classes of robots whose units cannot expand or contract.

Due to the interest of the tunneling capabilities for the purpose of reconfiguration, meta-modules of other robots have been designed that are able to expand and contract. Kotay and Rus (2000) have proposed an expandable and contractible meta-module for Molecules. More recently, Kawano (2020, 2019) has proposed a meta-module for slide-only cubes that can tunnel without disassembling. Murata and Kurokawa (2012) have presented a small and compact M-TRAN meta-module, although it can only expand and contract in two dimensions. In three dimensions, the only M-TRAN meta-module that we are aware of that can expand and contract is the one proposed by Aloupis et al. (2013), which is also valid for Molecubes (Zykov et al. 2007). Their meta-module is made of 58 units, and the length of the side of its minimum axis-aligned bounding box (cube) when expanded is 32 units. In addition to its size, its configuration is much less compact than the one by Murata and Kurokawa, making it less robust, in the sense that it has many degrees of freedom that are not necessary for the moves, but can make more difficult some actions such as the alignment between meta-modules when attaching to each other.

**Fig. 2:**
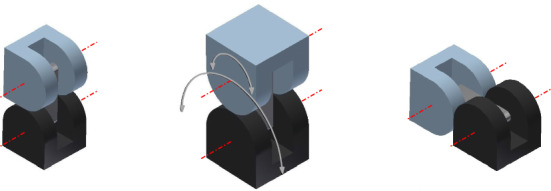
An edge-hinged unit and its degrees of freedom, as they appear in M-TRAN

The interest in the M-TRAN series of self-reconfiguring robots, from M-TRAN I to M-TRAN III (Kurokawa et al. 2008), is probably due to their simplicity and, at the same time, their versatility. M-TRAN is one of the geometrically fairest examples of a large class of so-called “edge-hinged” robots from a geometric viewpoint of its degrees of freedom (this class of robots is precisely defined in Sect. 2). Its units, depicted in Fig. 2, can be connected in chain or tree topologies and allow for continuous movement. This makes M-TRAN suitable for a variety of tasks and locomotion modes: among others, it can perform whole body locomotion as a crawler and as a traveling wave, as well as several legged gaits (Murata and Kurokawa 2012). At the same time, the M-TRAN units can be arranged in a cubic grid. In such a lattice architecture, it is simpler to plan reconfiguration. Molecubes or Roombots (Spröwitz et al. 2010) have a hinging mechanism that gives rise to a geometrically different rotation movement of their units, in what is known as “central-point-hinged” modular robots. Analogously to edge-hinged, central-point-hinged robot units can be arranged in chain, tree or lattice topologies as to produce different forms of reconfiguration.

### Contributions

- A geometric abstraction of two classes of modular robots that cover a wide set of existing modular systems, that we call *edge-hinged* and *central-point-hinged* modular robots. See Sect. 2.
- A new meta-module for edge-hinged modular robots that can expand and contract as to double/halve its size, just as Crystalline and Telecube units do. We prove that the meta-module is optimal in size and number of units. It is valid for M-TRAN (Kurokawa et al. 2008), SuperBot (Salemi et al. 2006), SMORES (Davey et al. 2012) UBot (Zhao et al. 2012), PolyBot (G3) (Yim et al. 2002) and CKBot (Park and Yim 2009). See Sect. 3.
- A new meta-module for central-point-hinged modular robots that can expand and contract as to double/halve its size, valid for Molecubes (Zykov et al. 2007) and Roombots (Spröwitz et al. 2010). The size of this meta-module is also optimal. See Sect. 4.
- Both meta-modules enable the application of tunneling reconfiguration algorithms for Crystalline and Telecube units (Aloupis et al. 2011; Butler and Rus 2003; Rus and Vona 2001; Vassilvitskii et al. 2002) to edge-hinged and central-point-hinged modular robots. However, these algorithms rely on meta-modules of $2\times 2 (\times 2)$ Crystalline or Telecube units that are able to perform three additional operations (*scrunch*, *relax*, and *transfer*). We further show that our meta-module for edge-hinged robots can also directly perform these operations. This implies that, to apply the constant-strength tunneling algorithms for Crystalline and Telecube units (Aloupis et al. 2011; Butler and Rus 2003; Vassilvitskii et al. 2002) to edge-hinged robots, meta-meta-modules are not necessary. As a side effect, the meta-module can also be used in surface traversing reconfiguration strategies based on the sliding-cube model. Thus, reconfiguration strategies as the ones illustrated in Fig. 1 can be directly applied to our meta-module for edge-hinged modular robots. See Sect. 5.

## Geometric abstraction of the units

In this section we define two geometric abstractions that allow us to model several current robot prototypes. First, we abstract the geometric properties of M-TRAN, and prove that our abstraction models a wide set of currently existing modular robots, including SuperBot, SMORES, CKBot, PolyBot, and UBot. We call this class *edge-hindged*. Then we abstract the geometric properties of Molecubes, and prove that our abstraction applies to Roombots too. We call this class *central-point-hinged*.

### Edge-hinged robot units

Our edge-hinged geometrical model is an abstraction of M-TRAN units. M-TRAN units consist of two linked semi-cylindrical cubes, as illustrated in Fig. 2. We refer to these two semi-cylindrical cubes as *blocks*. The minimum bounding box of each block is a cube. The units have two degrees of freedom: each semi-cylindrical block can rotate from $-\,90^{\circ }$ to $+\,90^{\circ }$ with respect to the link connecting both blocks. The semi-cylindrical shape of the blocks prevents self-intersections along the rotations. Each M-TRAN block has a gender (male/female) and connectors (different for the two genders) on its three flat surfaces. Two units can be attached through flat surfaces of different gender, in any of the four possible relative orientations.

We abstract the geometric properties of M-TRAN as follows.

1. Each unit of the robot consists of two blocks and a linkage between them.
2. The minimum bounding box of each block is a cube and each block is contained in a semi-cylindrical shape, union of half a cube and half a cylinder.
3. Each block can rotate from $-\,90^{\circ }$ to $+\,90^{\circ }$ with respect to the link joining both blocks.
4. Two units can be attached through their flat surfaces (with or without gender distinction), in any of the four possible relative orientations.

Following the terminology from Aloupis et al. (2013), we call the family of modular robots instantiating this geometric concept *edge-hinged*.

#### Observation 1

Although they have different technical peculiarities, M-TRAN, SuperBot, SMORES, CKBot U-bar, and PolyBot can fully instantiate edge-hinged modular robots. Moreover, Ubot and CKBot L7 can partially instantiate edge-hinged modular robots.

This is obvious for M-TRAN, since the edge-hinged model is a geometrical abstraction its properties. These geometric properties also apply to SuperBot (Salemi et al. 2006) units. The main geometric difference is that these units have an extra degree of freedom that is not required within the edge-hinged model (as illustrated in Fig. 3 the link can also rotate). Moreover, the connectors in this case are genderless.

**Fig. 3:**
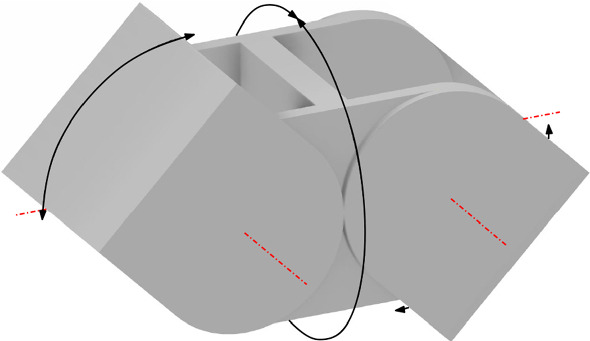
A SuperBot unit

One SMORES unit (Davey et al. 2012) consists of a U-shaped structure and a circular movable piece (see Fig. 4a) with 4 rotational degrees of freedom. Two SMORES units attached by their circular movable piece can behave like one Superbot unit. Therefore, SMORES can also instantiate an edge-hinged modular robot.

**Fig. 4:**
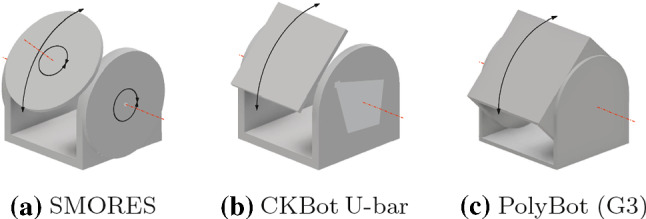
Illustration of the degrees of freedom of SMORES, CKBot U-bar, and PolyBot units

CKBots (Park and Yim 2009) include two kinds of units. U-bar units, illustrated in Fig. 4b, consist of a U-shaped structure and a square movable piece, but have only one degree of freedom. Nonetheless, two CKBot U-bar units, when attached by their square movable piece, have the same degrees of freedom as M-TRAN. Therefore, if they were equipped with self-reconfigurable connectors with the right parity, they could also instantiate an edge-hinged modular robot.

A PolyBot (G3) unit (Yim et al. 2002) consists of two U-shaped structures, one interior to the other, and has one degree of freedom (see Fig. 4c). When attached by their central face of the interior U-shaped structure, two units behave as in the previous case of CKBot U-bar units. Thus, PolyBot can also instantiate an edge-hinged modular robot.

A Ubot unit (Zhao et al. 2012) consists of two L-shaped parts connected by a 1-bend axis with two rotational degrees of freedom, illustrated in Fig. 5a. Two units of Ubot attached as shown in Fig. 5b can rotate as one M-TRAN unit or, more generally, as one edge-hinged unit. Notice that the faces perpendicular to the rotation axes in a edge-hinged unit do not behave as in two attached Ubot units. More precisely, one such face per Ubot unit behaves differently (in Fig. 5b the two edge-adjacent faces on the bottom). Nevertheless, this does not affect the moves we need for our expanding and contracting meta-module. A CKBot L7 (Park and Yim 2009) unit is geometrically equal to a Ubot unit, except for the fact that it only has one of the two rotational degrees of freedom of Ubots (see Fig. 5c). Thus, both Ubots and CKBot L7 partially instantiate edge-hinged modular robots.

**Fig. 5:**
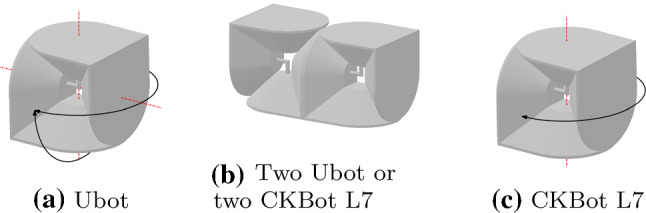
Illustration of the degrees of freedom of Ubot and CKBot L7 units, and how to attach two of them

In contrast, there are some seemingly very similar modular robots that does not fall into the edge-hinged model, as in the case of the iMobot (Ryland and Cheng 2010). The bounding box of an iMobot semi-cylindrical block is not a cube, but a right rectangular prism, due to its rotating faceplates (see Fig. 6). This difference has a huge significance when it comes to using the units of a modular robot in a lattice context since the units do no fit in a cubic lattice.

#### Observation 2

The modular robot iMobot does not instantiate the edge-hinged geometric model.

**Fig. 6:**
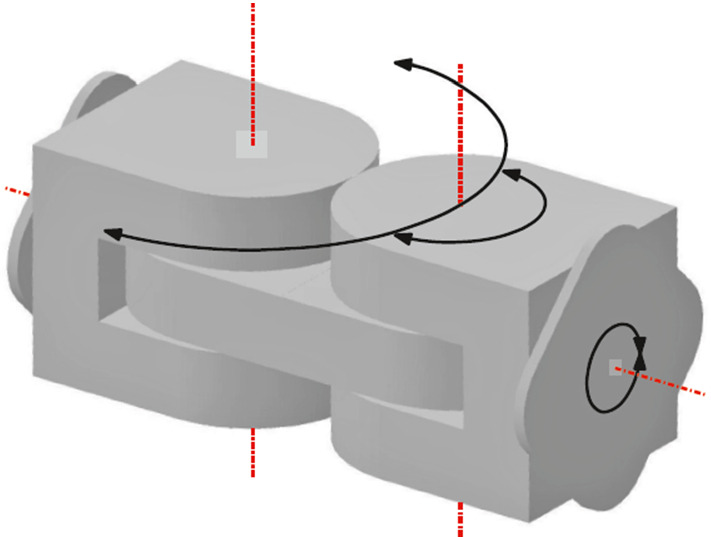
An i-Mobot unit

### Central-point-hinged robot units

Our central-point-hinged geometrical model is an abstraction of Molecube units. Molecube (Zykov et al. 2007) units are cube-shaped with rounded corners and two halves that can rotate relative to each other, giving rise to one degree of freedom. The two halves are created by partitioning the cube by a plane through its center and orthogonal to the line defined by two opposite vertices of the cube. Such line is the axis with respect to which the two halves of the unit can rotate, as shown in Fig. 7. The rounded corners are the result of intersecting the cube with a cylinder whose axis coincides with the rotation axis (see Fig. 8a). This prevents intersections of the rotating halves with adjacent units. All faces of a Molecube can connect in any possible orientation, with no gender distinction.

**Fig. 7:**
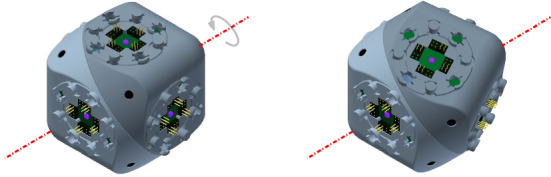
A central-point-hinged unit and its rotational degree of freedom, as it appear in Molecubes. Model from the Molecubes project (Zykov et al. 2007)

**Fig. 8:**
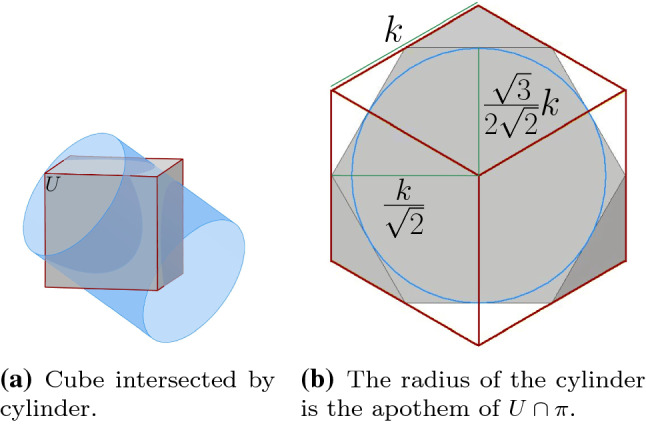
Construction of central-point-hinged units

We abstract the geometric properties of Molecubes as follows.

1. Each unit of the robot consists of one block whose minimum bounding box is a cube. Each block is divided into two halves by a plane through its center, orthogonal to the line defined by two opposite vertices of the minimum bounding cube (axis).
2. Each block is contained in the intersection of the minimum bounding cube and a cylinder whose axis coincides with the aforementioned axis, as to physically allow Property 3 in the presence of adjacent blocks. For this purpose, the appropriate radius of the cylinder is $\sqrt{3}\over 2\sqrt{2}$ times the length of the edges of the cubes (see Lemma 1).
3. Each half can rotate from $-\,120^{\circ }$ to $+\,120^{\circ }$ about the axis.
4. Two units can be attached through any of the six faces of their bounding cubes, in any of the four possible relative orientations. Notice that this abstraction is independent of whether the attachments have gender distinction or not.

Following the terminology from Aloupis et al. (2013), we call the family of modular robots instantiating this geometric concept *central-point-hinged*.

#### Observation 3

Although they have different technical peculiarities, Molecube and Roombots can instantiate central-point-hinged modular robots.

Since the central-point-hinged model is a geometrical abstraction of Molecube units, we only need to argue that they also model Roombots (Spröwitz et al. 2010). Each Roombot unit is made of two rounded cubes that are divided into two halves in the same way Molecubes are. Each half can continuously rotate about the axis, and the faces of the cube can attach and detach from neighboring modules in all directions, with no gender restrictions. In fact, a Roombot unit has one extra rotational degree of freedom between its two blocks (see Fig. 9). Therefore, one unit of Roombots can behave as one Molecube 2-unit chain.

**Fig. 9:**
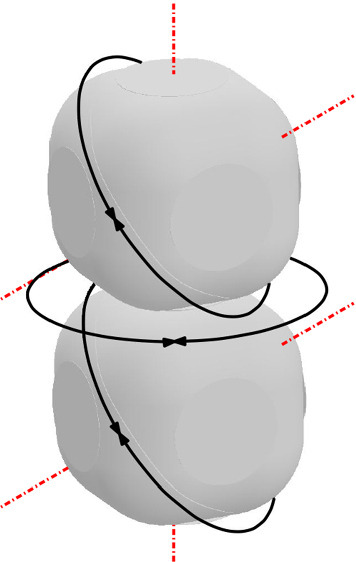
A Roombot unit

**Fig. 10:**
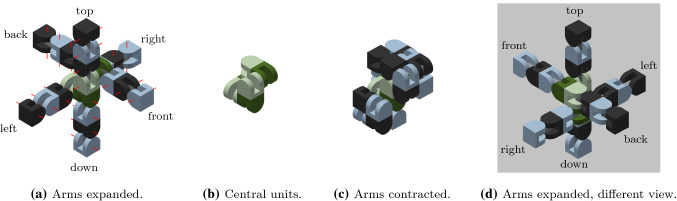
Meta-module for edge-hinged units

#### Lemma 1

In order to allow full rotation of one half-unit of a central-point-hinged robot in the presence of neighboring units, it is necessary and sufficient that the bounding cylinder of the unit has radius $\sqrt{3}\over 2\sqrt{2}$ times the length of the edges of the cubes.

#### Proof

Let *u* and $u'$ be the two opposite vertices of the bounding cube *U* of a central-point-hinged unit along its rotation axis, and let $\pi$ be the perpendicular bisecting plane of segment $uu'$. Plane $\pi$ separates the two halves of the unit.

The intersection $U\cap \pi$ is a regular hexagon whose edges are the intersection of $\pi$ with the six neighboring lattice cells of *U*. Therefore, if the unit was a cube, even an infinitesimal rotation of a half of the unit would mean invading its neighboring lattice cells. Free rotation within the lattice cell is possible if and only if the unit is contained in the cylinder of axis $uu'$ whose radius equals the apothem of the hexagon (i.e., the length of a line segment from the center of the hexagon to the midpoint of one of its sides). Refer to Fig. 8b. If *k* is the length of any edge of *U*, then the hexagon sides have length $k/\sqrt{2}$, and its apothem has length ${\sqrt{3}\over 2\sqrt{2}}k$. $\square$

## Our meta-module design for edge-hinged modular robots

In this section we describe how edge-hinged units can be combined into a meta-module that is able to expand and contract. We will prove its correctness in the edge-hinged model and it implies it for M-TRAN, SuperBot, SMORES, UBot, PolyBot, and CKBot.

### Description of the meta-module

The proposed meta-module for edge-hinged units, illustrated in Fig. 10a, consists of 6 *arms*, aligned in three directions that are parallel to the *x*, *y* and *z* axes.

**Fig. 11:**
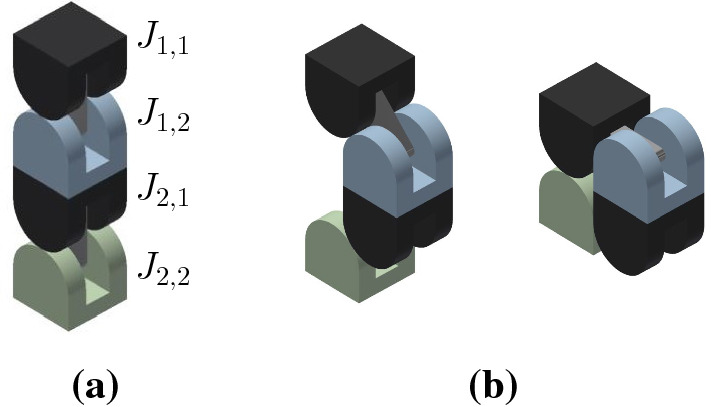
Each arm is implemented using a 2-unit chain. **a** The four semi-cylindrical blocks of the top 2-unit chain of the top-down 4-unit chain from Fig. 10a labelled $J_{1,1}$ (tip), $J_{1,2}$, $J_{2,1}$, and $J_{2,2}$ (central). **b** The edge-hinged arm is able to contract while maintaining its potential connections at both ends

Each arm is implemented using a 2-unit chain. It consists of two units attached at square flat faces, with the direction of their links aligned, as shown in Fig. 11.

**Fig. 12:**
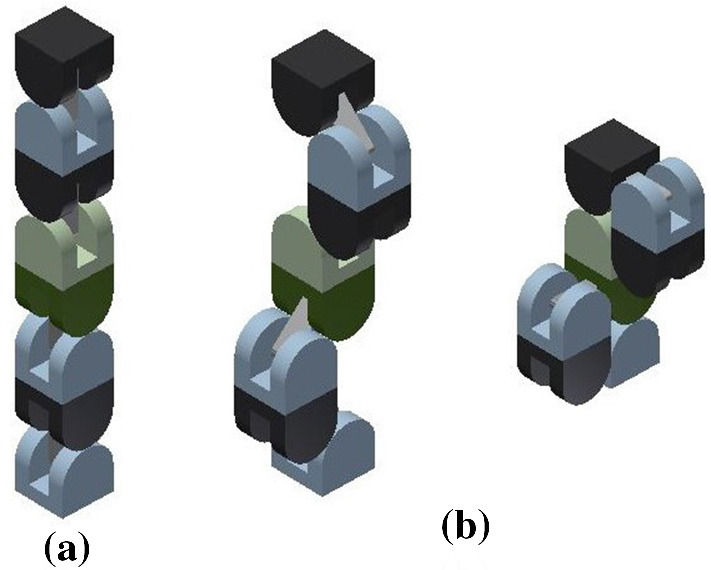
**a** Connecting two edge-hinged arms into a 4-unit chain. The central blocks are highlighted in green. **b** The contraction movement of one 4-unit chain (Color figure online)

For each of the three directions parallel to the *x*, *y* and *z* axes, two such arms are connected to each other, resulting in a 4-unit chain whose blocks are all aligned. However, the linkages of the two connected arms differ in their orientations (see Fig. 12). This design decision is important in order to endow the meta-module with as much mobility as possible. We call the blocks connecting the two arms *central*. The end blocks of a 4-unit chain we call *tips*.

Let us introduce some notation before we describe the meta-module in more detail. We number from 1 to 4 the four units of any of the 4-unit chains, as they appear along the chain, in some order that we will precise when necessary. We denote by $J_{i,1}$ and $J_{i,2}$ the two blocks of the *i*-th unit, as they appear in the same order.

The meta-module is formed by connecting three 4-unit chains, one for each of the *x*, *y* and *z* directions. The connection is done through their central blocks (see Fig. 10b) at their semicircular faces. We note that each central block has only one connection through a semicircular face. This implies that the meta-module also applies to robots like Ubot and CKBot L7 that partially instantiate edge-hinged modular robots. Fig. 10a, d show the meta-module with all its arms expanded and Fig. 10c shows it with all its arms contracted.

To describe the meta-module more precisely, we will refer to the three 4-unit chains as *top-down*, *back-front*, and *left-right* (refer to Fig. 10a), and we will number their units in that same order. Then, the attachments between the three chains are done as follows: $J_{2,2}$ of the top-down chain is attached to $J_{3,1}$ of the back-front chain. Analogously, $J_{2,2}$ of the back-front chain is attached to $J_{3,1}$ of the left-right chain. Finally, $J_{2,2}$ of the left-right chain is attached to $J_{3,1}$ of the top-down chain.

### Meta-module’s properties

The goal of this design is to obtain a minimum-size meta-module that can perform the Crystalline and Telecube unit operations expand, contract, attach and detach. To this aim, the meta-module should have the following key properties:

1. Each arm is able to independently expand and contract, doubling and halving its length, respectively. Moreover, during the move of an arm, the six central blocks are kept still, while the two tips of the arm stay aligned and maintain their orientations. This last property is critical, as it guarantees the connectivity with the rest of the robot in reconfiguration algorithms. This will be proved in Lemma 2.
2. When arms are moving, no arm collides with any other part of the meta-module. This will be proved in Lemma 3.
3. Throughout the move, the robot stays connected. This will be proved in Lemma 4.
4. The size of the meta-module is minimal. This will be proved in Theorem 5.

In the next section we formalize and prove the properties above.

### Proof of the meta-module’s properties

All the following properties are stated in terms of the contraction move. It is straightforward to realize that they also hold for the expansion move, which is no more than the reverse of a contraction.

We start proving a key property of the arms. Namely, that the rotation of the blocks allows each arm to contract an expand, while preserving potential connections.

#### Lemma 2

The edge-hinged arm of the meta-module can be contracted. Throughout the move its two extremal blocks (central and tip) stay aligned and keep their orientation, while the centers of the four blocks always lie in the same plane. By the end of the contraction, the distance between the extremal faces of the central and tip blocks of the arm is half the distance when expanded.

#### Proof

Without loss of generality, let us discuss the case of a 2-unit chain $J_{1,1}$,$J_{1,2}$,$J_{2,1}$, and $J_{2,2}$. The proof for $J_{3,1}$,$J_{3,2}$,$J_{4,1}$, and $J_{4,2}$ is symmetric.

$J_{1,1}$ and $J_{1,2}$ are connected through a rotational link, and so are $J_{2,1}$ and $J_{2,2}$. A rigid attachment exists between $J_{1,2}$ and $J_{2,1}$.

The contraction, shown in Fig. 11, consists of a $-90^{\circ }$ rotation of $J_{1,1}$ and $J_{2,2}$ and a $+90^{\circ }$ rotation of $J_{1,2}$ and $J_{2,1}$ about their respective links. The realization of this move is allowed by the two rotational degrees of freedom and the semi-cylindrical shape of the blocks.

Notice that the two rotation axes of an arm are parallel. By construction, their direction is perpendicular to that of the line connecting all the centers of the blocks when the arm is in extended position. Therefore, the centers of the blocks stay in the same plane throughout the contraction move. Furthermore, the contraction does not change neither the alignment nor the orientation of the central and tip blocks of the chain, $J_{1,1}$ and $J_{2,2}$, due to the opposite sign of the rotations applied to each pair of blocks connected by a rotational link. Indeed, since $J_{2,2}$ is central and stays still, then the composition of the four rotations gives rise to a translation of $J_{1,1}$ in the direction of the arm, towards $J_{2,2}$. Notice that the requirement for this statement to be true is that the move consists of a rotation of angle $-\alpha$ for $J_{1,1}$ and $J_{2,2}$, and a rotation of angle $+\alpha$ for $J_{1,2}$ and $J_{2,1}$ about their respective links, for $\alpha$ going from $0^\circ$ to $90^\circ$. In other words, the property that the two rotation angles used are the same with opposite signs must be maintained throughout the entire contraction.

It is easy to prove that no self-intersection of the arm happens throughout the contraction. Blocks within the same unit cannot self-intersect by construction. On the other hand, $J_{1,2}$ and $J_{2,1}$ cannot self-intersect since they are rigidly attached to each other. In order to prove that $J_{1,1}$ can neither intersect $J_{2,1}$ nor $J_{2,2}$, let us assume, without loss of generality, that the arm is aligned parallel to the *x* axis, and the minimum bounding box of each of its blocks has size $1\times 1\times 1$. Let $x_1$, $x_2$, $x_3$, and $x_4$ be the *x*-coordinates of the centers of the bounding boxes of $J_{1,1}$, $J_{1,2}$, $J_{2,1}$, and $J_{2,2}$ respectively. It is immediate to realize that $x_1\le x_2$, $x_3=x_2+1$, and $x_3\le x_4$ throughout the contraction, due to the size of the units and the rotation axes and angles of the blocks. Therefore, $x_4\ge x_3 \ge x_1+1$. This implies that $J_{1,1}$ can never intersect $J_{2,1}$ nor $J_{2,2}$, since the minimum bounding boxes of the blocks stay aligned and never change their orientation throughout the move. By symmetry, this also proves that $J_{2,2}$ can neither be intersected by $J_{1,2}$ nor by $J_{1,1}$ throughout the contraction.

Finally, since the minimum bounding box of each block is a cube, by the end of the contraction the distance between the extremal faces of the chain has been halved. $\square$

Next we show that contracting and expanding the arms of the meta-module does not produce collisions. We will use the fact that the linkages of the two arms forming a 4-unit chain have different directions. Therefore, their contraction and expansion movements take place in two orthogonal planes, as illustrated in Fig. 12 right.

#### Lemma 3

No self-intersection is produced when contracting any arms of the edge-hinged meta-module.

#### Proof

**Fig. 13:**
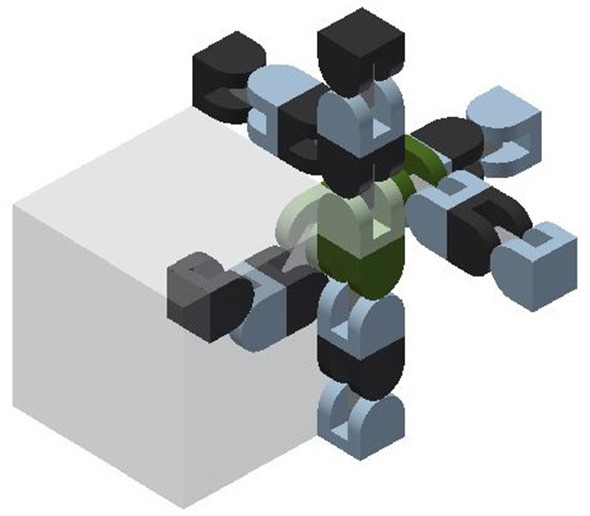
Edge-hinged meta-module contracting an arm in its corresponding octant

Consider the minimum axis-aligned cube containing the expanded meta-module and decompose it into eight octants through its center. By construction, each expanded arm is contained in a different octant (and two octants are empty). By Lemma 2, the centers of the four blocks of a contracting arm always lie in the same plane, so that the contraction of an arm sweeps a sub region of its corresponding octant (see Fig. 13). This guarantees that collisions between arms cannot occur. Collisions within one arm are excluded by Lemma 2. $\square$

**Fig. 14:**
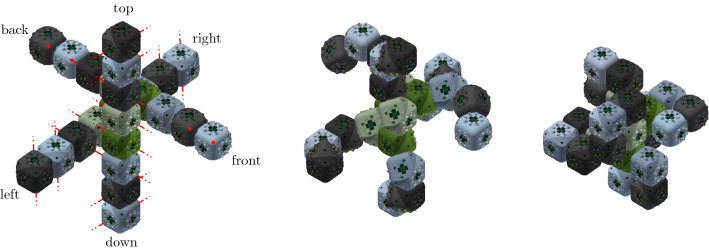
Meta-module for central-point-hinged units. From left to right: expanded, contracting (60$^{\circ }$), contracted

Finally, we prove that expanding and contracting arms can be done without disconnecting the robotic system.

#### Lemma 4

Throughout the contraction of any subset of arms of an edge-hinged meta-module, the robot stays connected.

#### Proof

While contracting any arm, its central block does not change its orientation (see Lemma 2). Hence, it does not need to change its attachments. Therefore, the six central blocks can maintain the meta-module connected at all times. Moreover, connectivity with neighboring meta-modules is preserved: if the tip of an arm is attached to the tip of another meta-module arm, Lemma 2 guarantees that this attachment can be maintained throughout expansion and contraction. $\square$

From the previous lemmata we conclude that the length of the meta-module can be reduced by half (when expanded arms are contracted) or doubled (when contracted arms are expanded) in any of the *x*, *y* and *z* directions. This can be done while preserving connectivity (Lemma 4) and avoiding collisions (Lemma 3). Attachments and detachments of the meta-module are performed by the tips of its six arms.

#### Theorem 4

The edge-hinged meta-module can perform the Crystalline and Telecube unit operations: expand, contract, attach, and detach.

Consider a lattice such that in the expanded configuration of the meta-module each of the edge-hinged blocks fits in a unit cell. We define the *size* of the meta-module as the length of its minimum bounding cube. In other words, the size of the meta-module measures the resolution of any configuration of meta-modules.

#### Theorem 5

The edge-hinged meta-module has optimal size.

#### Proof

Since the meta-module must be able to contract to fit in a cube of half side length, its size has to be even, both in its expanded and in its contracted form. Otherwise, it will be unable to align in the grid, and therefore with its neighbors. As the size of our meta-module is eight, the only remaining option is size four.

In this case, notice that the compressed meta-module would have only $2\times 2 \times 2 = 8$ cells. However, any meta-module of size four requires at least five edge-hinged units, which occupy ten lattice cells. Indeed, the meta-module needs to connect the six faces of the bounding cube. Therefore, since parallel external faces of the bounding cube are at distance four, the meta-module must contain a subtree with three connections in each of the three directions *x*, *y*, and *z* of the cube. These nine different connections imply that the meta-module must occupy at least ten cells. $\square$

## Extension to the central-point-hinged case

In this section we extend the previous results to central-point-hinged units. In particular, they will apply to Molecubes and Roombots.

### Description of the meta-module

The meta-module for central-point-hinged units is similar to the one described in Sect. 3. When expanded, it also consists of six arms aligned in the *x*, *y* and *z* directions (see Fig. 14).

Each arm consists of a chain of four units (i.e., 4 Molecube units or 2 Roombot units). If we number them, 1, 2, 3, and 4, as they consecutively appear in some order, we denote the two halves of the *i*-th unit as $J_{i,1}$ and $J_{i,2}$, as they appear in the same order. The plane separating them is denoted $\pi _i$, and the rotation axis $\ell _i$. The center of the bounding cube of the *i*-th unit $U_i$ is $O_i=(x_i,y_i,z_i)$.

**Fig. 15:**
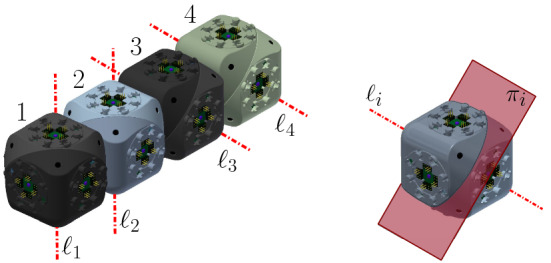
The central-point-hinged arm. The rotation axes and the plane separating the two halves of a unit are shown in red (Color figure online)

The rotation axes $\ell _1$ and $\ell _2$ are parallel, and so are $\ell _3$ and $\ell _4$. In addition, the rotation axes $\ell _2$ and $\ell _3$ share a point, namely one of the common vertices of the minimum bounding cubes of $U_2$ and $U_3$. We refer to $J_{1,1}$ as the *tip* half-unit of the arm, and to $J_{4,2}$ as *central* (see Fig. 15 for an illustration).

**Fig. 16:**
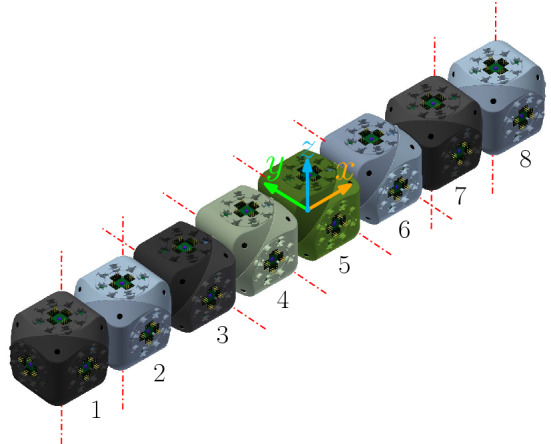
Connecting two arms in the same direction. The rotation axes are shown in red (Color figure online)

Similarly to the edge-hinged case, for each of the three directions parallel to the *x*, *y* and *z* axes, two such arms are connected to each other, through their central half units, forming a chain whose eight units are all aligned (see Fig. 16 for an illustration). If we number from 1 to 8 the units of the resulting 8-unit chain as they appear in consecutive order, the attachment is done such that the four rotation axes $\ell _3$, $\ell _4$, $\ell _5$, and $\ell _6$ are all parallel, and so are $\ell _1$, $\ell _2$, $\ell _7$, and $\ell _8$.

Let us describe in more detail the case where the two connected arms are parallel to the *x* axis (refer again to Fig. 16). Assume that each arm is made out of units whose minimum bounding box has size $1\times 1\times 1$. One of the arms is located in the octant $x\le 0$, $y\ge 0$, $z\le 0$. Let us label its units from 1 to 4. Then one vertex of $U_4$ is positioned at the origin and one edge of each of the four arm units lie in the *x* axis. The rotation axes $\ell _2$ and $\ell _3$ of this first arm are located as to share point $(-2,1,0)$. The second arm parallel to the *x* axis is attached to the first one at the plane $x=0$, such that it lies in the *x*-opposite octant $x\ge 0$, $y\ge 0$, $z\le 0$. The attachment is done such that $\ell _6$ and $\ell _7$ of this arm share point $(2,0,-1)$. This orientation structure is reproduced in the *y* and *z* directions.

The design of the meta-module connects each 8-unit chain through its central half-unit to the central half-unit of the remaining two 8-unit chains, similarly to what was done for the edge-hinged case (see Fig. 14). More precisely, if the numbering of the units is done top to down, back to front, and left to right, the attachment between them in the meta-module are as follows: $J_{4,2}$ of the top-down chain is attached to $J_{5,1}$ of the back-front chain. Analogously, $J_{4,2}$ of the back-front chain is attached to $J_{5,1}$ of the left-right chain. Finally, $J_{4,2}$ of the left-right chain is attached to $J_{5,1}$ of the top-down chain.

In summary, the 8-unit chain parallel to the *x* axis lies in the quadrant $y\ge 0$, $z\le 0$, all its units have one edge in the *x* axis, $\ell _2$ and $\ell _3$ share point $(-2,1,0)$, and $\ell _6$ and $\ell _7$ share point $(2,0,-1)$. Analogously, the 8-unit chain parallel to the *y* axis lies in the quadrant $x\ge 0$, $z\ge 0$, all its units have one edge in the *y* axis, $\ell _2$ and $\ell _3$ share point (0, 2, 1), and $\ell _6$ and $\ell _7$ share point$(1,-2,0)$. Finally, the 8-unit chain parallel to the *z* axis lies in the quadrant $x\le 0$, $y\le 0$, all its units have one edge in the *z* axis, $\ell _2$ and $\ell _3$ share point $(-1,0,2)$, and $\ell _6$ and $\ell _7$ share point$(0,-1,-2)$.

### Meta-module’s properties

The goal of this design is to obtain a minimum-size meta-module that can perform the Crystalline and Telecube unit operations expand, contract, attach and detach. The key properties for this meta-module are essentially the same four as for the edge-hinged case. However, some of the proofs become more involved, since now the movement of each arm does not take place in a fixed plane. We prove each of them in the next section.

### Proof of the meta-module’s properties

We start by defining more formally the the contract/expand move for one single arm. Without loss of generality, we will discuss the details for the case of an extended arm parallel to the *x* axis, in the octant $x\ge 0$, $y\ge 0$, $z\le 0$. Recall that the rotation axes of this arm, $\ell _6$ and $\ell _7$ are located as to share point $(2,0,-1)$.

Let us orient positively the rotation axes of all the arm units towards $(2,0,-1)$. The contraction move consists of simultaneously rotating $-120^{\circ }$ both $J_{5,2}$, $J_{6,1}$, $J_{7,1}$, and $J_{8,2}$ about their respective axes, while $J_{5,1}$, $J_{6,2}$, $J_{7,2}$ and $J_{8,1}$ rotate $+120^{\circ }$ about theirs. The realization of the contraction move is allowed by the one rotational degree of freedom of each unit.

**Fig. 17:**
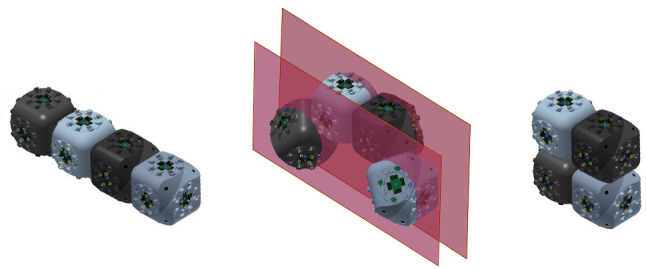
Contraction of a central-point-hinged arm

This move (illustrated in Fig. 17) can alternatively be described in the following way. Let us start by looking at the 5th and 6th units, $U_5$ and $U_6$. Since $J_{5,1}$ is attached to the remaining arms of the module, it stays still. Therefore, $J_{5,2}$ rotates $-120^{\circ }$ relative to $J_{5,1}$, about $\ell _5$. Since $J_{6,1}$ stays rigidly attached to $J_{5,2}$, it also rotates $-120^{\circ }$ about $\ell _5$, carrying $\ell _6$ with it. Finally, $J_{6,2}$ rotates $+120^{\circ }$ (relative to $J_{6,1}$) about $\ell _6$.

The first thing to be noticed is that, in the central-point-hinged case, the expansion and contraction of each arm does not occur in a plane, as opposed to what happens in the edge-hinged case. The central image in Fig. 17 illustrates this. As a consequence, the shapes of the two meta-modules differ along the contraction. Furthermore, the proofs of the results analogous to Lemmas 2 and 3 get more involved than in the central-point-hinged case.

**Fig. 18:**
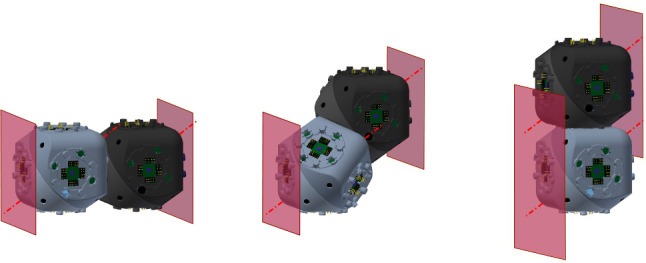
While contracting a central-point-hinged arm, units $U_5$ and $U_6$ are confined between planes $\pi _0$ and $\pi _{6,7}$ at all times

We start by analyzing the move of the two adjacent units of half one arm.

#### Lemma 5

Consider an extended arm parallel to the *x* axis, in the octant $x\ge 0$, $y\ge 0$, $z\le 0$. Let $\pi _0$ be the plane $x=0$, and let $\pi _{6,7}$ be the plane supporting the cube facet of $U_6$ common to $U_7$. Throughout the contraction move, $\pi _{6,7}$ moves parallel to itself from $x=2$ to $x=1$, and $U_5$ and $U_6$ always lie between $\pi _0$ and $\pi _{6,7}$ (see Fig. 18).

#### Proof

When the arm is extended, $\pi _{6,7}$ is the plane $x=2$. Let us start proving that, as the arm contracts, $\pi _{6,7}$ moves parallel to itself.

When the arm starts in extended position, $\ell _6$ is parallel to $\ell _5$. Throughout the move, $\ell _6$ rotates about $\ell _5$. Therefore, it stays parallel to $\ell _5$ at all times. Since the two $\pm 120^{\circ }$ rotation angles have opposite signs, we obtain that $J_{6,2}$ does not change orientation: it moves parallel to itself throughout the arm contraction, and so does $\pi _{6,7}$. In fact, $\pi _{6,7}$ occupies the positions $x=f(\alpha )={4\over 3}+{2\over 3}\cos \alpha$ where $\alpha$ is the rotation angle of $J_{5,2}$ about $\ell _5$, decreasing from $0^{\circ }$ to $-120^{\circ }$.

Let us now prove that $U_5$ lies between planes $\pi _0$ and $\pi _{6,7}$ throughout the contraction. Trivially, $J_{5,1}$ stays between $\pi _0$ and $\pi _{6,7}$ at all times, since it does not move, and $\pi _{6,7}$ monotonically moves parallel to itself from $x=2$ to $x=1$. Consider now $J_{5,2}$. As it rotates about $\ell _5$, it stays within the cylinder that has $\ell _5$ as axis and contains $U_5$. Therefore, it always lies to the right of $\pi _0$. Furthermore, as long as the absolute value of the rotation angles stays between $0^{\circ }$ and $120^{\circ }$, $J_{5,2}$ cannot intersect $\pi _{6,7}$ either. Indeed, even if $J_{5,2}$ had not been intersected with the cylinder, and was simply a half-cube, it would not intersect $\pi _{6,7}$, since the *x*-coordinate of its most extreme vertex (1, 1, 0) would follow the trajectory given by the equation $x=g(\alpha )={1\over 3\sqrt{3}}(2\sqrt{3}+\sqrt{3}\cos \alpha -3\sin \alpha )$, which is obtained by applying to the vertex a rotation of angle $\alpha$ about $\ell _5$. But we have $f(\alpha )> g(\alpha )$ for all $\alpha \in (-120^{\circ },0^{\circ }]$, and $f(-120^{\circ })\ge g(-120^{\circ })=1$.

The proof that $U_6$ also stays between planes $\pi _0$ and $\pi _{6,7}$ throughout the contraction move is symmetrical, by considering all the moves relative to $J_{6,2}$. $\square$

#### Lemma 6

The central-point-hinged arm can be contracted. Throughout the move its two extremal (central and tip) halves stay aligned and keep their orientation. By the end of the contraction, the distance between the extremal faces of the central and tip units of the arm is half the distance when expanded.

#### Proof

We already know that Lemma 5 applies to $U_5$ and $U_6$. The analogous symmetric statement holds for $U_7$ and $U_8$. Since $J_{7,1}$ is rigidly attached to $J_{6,2}$, we can conclude the following:

- $J_{6,2}$ and $J_{7,1}$ move parallel to themselves without changing orientation throughout the arm contraction.
- $J_{8,2}$ stays aligned with $J_{5,1}$ at all times. In other words, it undergoes a translation by a vector parallel to the *x* axis, towards $J_{5,1}$.
- The arm halves its length in the direction of the *x* axis.
- No self-intersection of the arm happens throughout the contraction.

The first two statements are true because of the opposite signs and equal absolute value ($120^{\circ }$) of the rotations. The third statement is a direct consequence of Lemma 5.

**Fig. 19:**
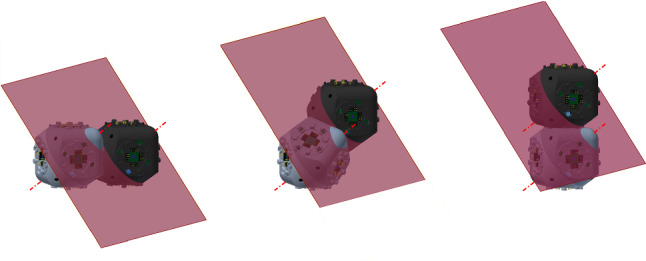
Throughout the contraction, the plane $\pi _6$ keeps $J_{6,2}$ away from $J_{6,2}$ and $U_5$

In order to prove the forth and last statement, we start by recalling that the plane $\pi _{6,7}$ supporting the cube facet connecting $U_6$ and $U_7$ separates the first half of the arm (i.e., $U_5$ and $U_6$) from the second half ($U_7$ and $U_8$) throughout the move. Therefore, we only need to prove that $U_5$ and $U_6$ cannot collide. Recall that $J_{5,2}$ and $J_{6,1}$ are rigidly attached throughout the contraction move. This implies that they cannot collide. Furthermore, it also implies that $\pi _5$ and $\pi _6$ stay parallel and keep their distance constant at all times. Therefore, $J_{5,1}$ stays separated from $J_{5,2}$ and $U_6$ by $\pi _5$ throughout the entire move. Symmetrically, $J_{6,2}$ stays separated from $J_{6,1}$ and $U_5$ by $\pi _6$. Figure 19 shows this separation property.

Since $U_5$ and $U_6$ do not intersect, by symmetry $U_7$ and $U_8$ do not intersect either. $\square$

As in the edge-hinged case, the centers of the four units of a central-point-hinged arm are coplanar at all times during a contraction move. Nevertheless, in contrast to the edge-hinged case, such plane does not stay still throughout the move, as shown in Fig. 17. This makes the proof of the following lemma—analogous to Lemma 3—more difficult for the central-point-hinged case.

#### Lemma 7

No self-intersection is produced when expanding or contracting any arms of the central-point-hinged meta-module.

#### Proof

Consider the minimum axis-aligned cube containing the expanded meta-module and decompose it into eight octants through its center. In what follows, we stick to the position of the metamodule described in Sect. 4. It is easy to see that each expanded arm is contained in a different octant. Two centrally opposite octants are empty, namely $x\ge 0$, $y\ge 0$, $z\ge 0$ and $x\le 0$, $y\le 0$, $z\le 0$. Note that each octant containing an arm shares two faces with octants that also contain arms, and one face with an empty octant.

First, we argue that the six octants containing one arm each are not intersected by any other arm during the contraction. We start by noticing that $J_{5,1}$ is fixed. Symmetrically, $J_{8,2}$ is translated towards $J_{5,1}$ along a direction parallel to the arm’s coordinate axis. Therefore, none of them ever exits their initial octant. It is easy to see that $U_6$ and $U_7$ move always inside the octant. More precisely, $J_{6,1}$ and $J_{7,2}$ rotate towards the interior of the octant, making $J_{6,2}$ and $J_{7,1}$ move parallel to themselves within the octant. Finally, while rotating, $J_{5,2}$ and $J_{6,1}$ do not invade any of the two neighboring octants containing arms. The reason being that they rotate within a half cylinder that does not intersect those octants. By Lemma 6, no collisions occur within these octants.

Each of the two empty octants, though, are intersected by the rotating halves $J_{5,2}$ and $J_{8,1}$ (or $J_{1,2}$ and $J_{4,1}$) of three different arms. The halves $J_{1,2}$ and $J_{8,1}$ cannot produce collisions, as they invade the empty octant close to their corresponding coordinate axis, and only in the interval between 4 and 2 (or $-4$ and $-2$), i.e., far away from each other. It remains to verify that no collisions occur between the halves $J_{4,1}$ or $J_{5,2}$. This is due to the fact that each such half unit rotates inside its corresponding cylinder, and these cylinders are all parallel and pairwise disjoint. $\square$

Using proofs analogous to those of Lemma 4, Theorem 4, and Theorem 5 in the previous section (replacing Lemmas 2, 3, and 4 by Lemmas 6, 7, and 8, respectively), we obtain the following statements.

#### Lemma 8

Throughout the contraction move of any subset of arms of a central-point-hinged meta-module, the robot stays connected.

#### Theorem 6

The central-point-hinged meta-module can perform the Crystalline and Telecube unit operations: expand, contract, attach, and detach.

Finally, the meta-module in this case is also as small as possible.

#### Theorem 7

The central-point-hinged meta-module has optimal size.

## Avoiding meta-meta-modules

**Fig. 20:**
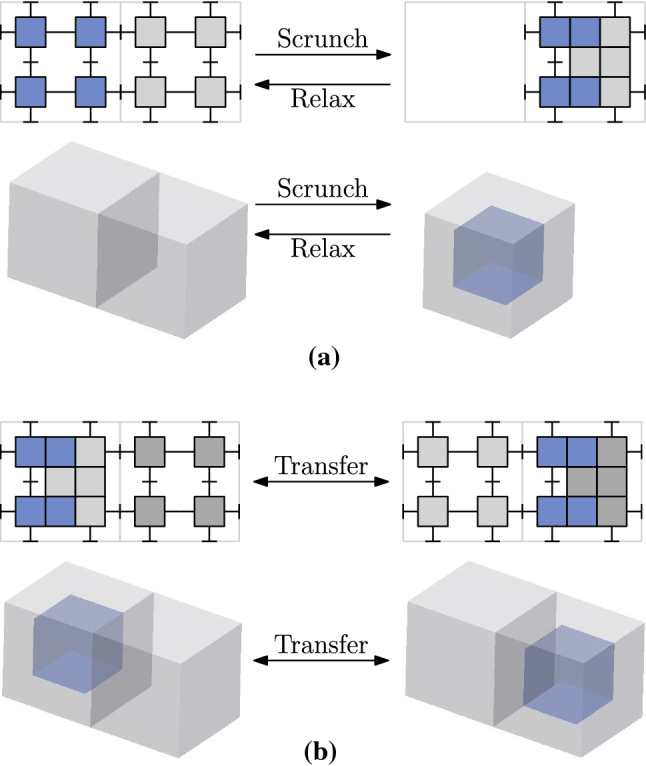
Crystalline and Telecube meta-module scrunch and relax (**a**), and transfer (**b**). Top: actual position of the Crystalline and Telecube units in dimension 2. Bottom: symbolic representation in dimension 3

By Theorem 4, we can apply tunneling reconfiguration algorithms for Crystalline and Telecube units (Aloupis et al. 2011; Butler and Rus 2003; Rus and Vona 2001; Vassilvitskii et al. 2002) to our meta-module. These algorithms, in turn, use meta-modules of $2\times 2(\times 2)$ Crystalline or Telecube units that are able to perform the following operations:

- *Scrunch* and *Relax*: compressing two neighboring connected meta-modules so that both occupy the same single lattice cell, and the reciprocal operation (see Fig. 20a).
- *Transfer*: a compression in a meta-module is transferred to an adjacent lattice cell whose meta-module is not compressed (see Fig. 20b).

In Sect. 3 we have shown that the new meta-module is able to perform the expand and contract Crystalline and Telecube unit operations. In this section we show that for the case of edge-hinged modular robots, it is also able to perform scrunch, relax, and transfer. This decreases the resolution of the configurations that are needed, both in size and number of units, since the reconfiguration algorithms can be applied without the need of meta-meta-modules of edge-hinged units. Moreover, it also reduces the coordination and synchronization needed to implement the reconfiguration algorithms.

**Fig. 21:**
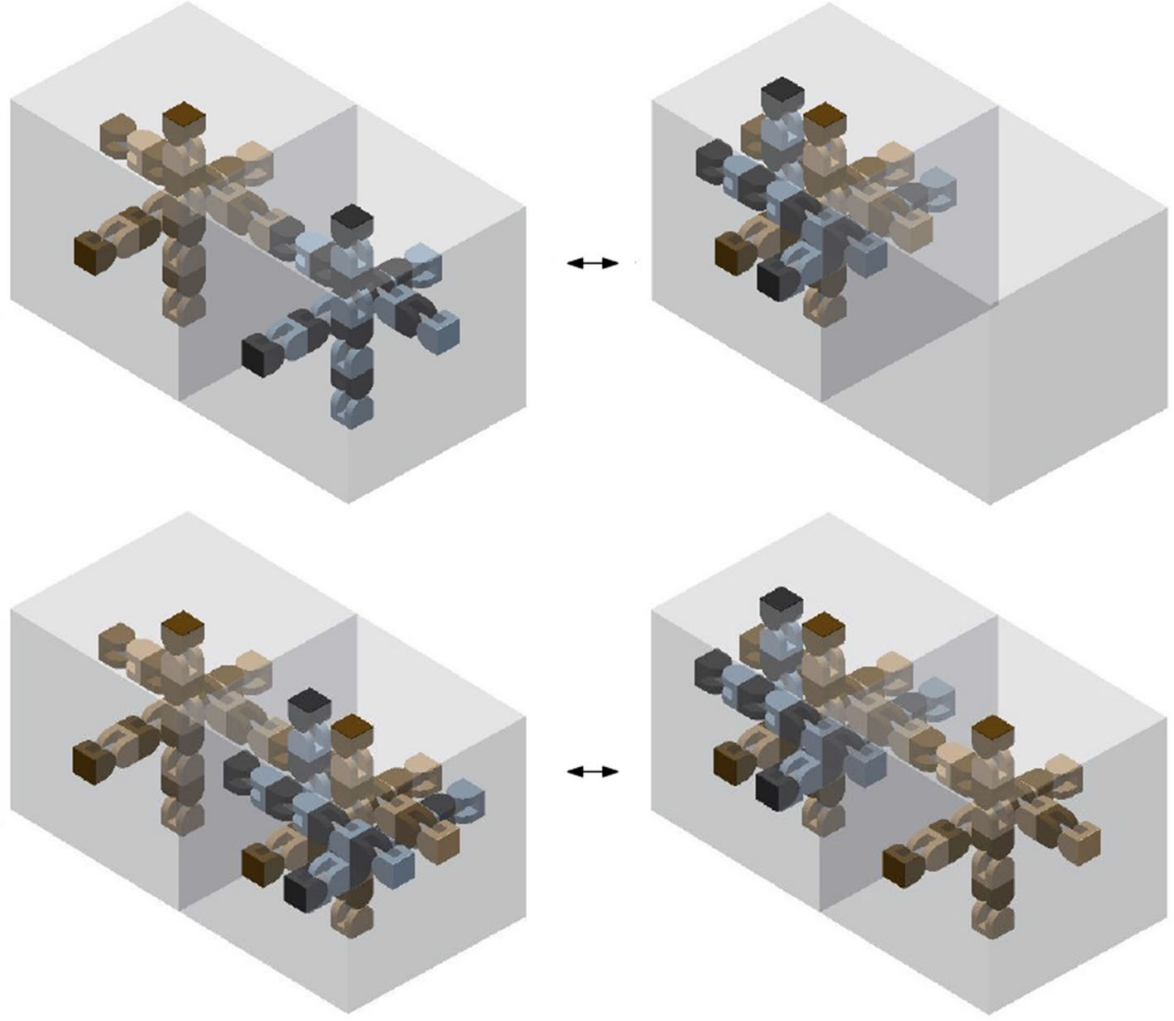
Top: the scrunch/relax operation. Bottom: the transfer operation. Notice the canonical position of the blue meta-module when compressed (Color figure online)

We start by describing how two meta-modules can occupy one single lattice cell in a *canonical* position. One of the two meta-modules, that we will call *host*, is positioned in the standard way defined in Sect. 3, guaranteeing the connectivity of the overall structure. The other meta-module, that we will call *guest*, is positioned as follows (see Fig. 21):

1. The three 4-unit chains of the guest meta-module are parallel to those of the host. Also their linkages are parallel to those of the host.
2. Each guest 4-unit chain stands at lattice distance 1 from its parallel host 4-unit chain.
3. Guest 4-unit chains are attached through their central blocks to a perpendicular host 4-unit chain.

We are now ready to prove that our meta-module is able to perform scrunch, relax and transfer. In a scrunch/relax, the host meta-module stays still, while the other one becomes its guest (scrunch) or leaves the guest position and locates itself in a neighboring empty lattice position (relax). In a transfer, two adjacent host meta-modules stay still, while the guest moves from the canonical position attached to one of the host meta-modules to the canonical position attached to the other one.

This leads to the following result.

### Theorem 8

The edge-hinged meta-module can perform the Crystalline and Telecube meta-module operations scrunch/relax and transfer.

### Proof

Both scrunch/relax and transfer can be obtained by only moving the scrunching, relaxing or transferred meta-module, while all the remaining elements of the robot configuration stay still. More precisely, each 4-unit chain of the moving meta-module is able to move in all *x*, *y* and *z* directions as to reach its final destination, which is parallel to its initial one. The symmetry of the meta-module together with that of the canonical position for two meta-modules within one lattice cell, allows to perform scrunch/relax and transfer on the moving meta-module to place it in any of the six adjacent lattice cells.

Furthermore, the moves can be planned so that no collision occurs. Indeed, even when several rotations are performed in parallel, the moving arm never collides with other arms (of the guest or the host meta-modules), nor it exceeds the boundary of the union of the two lattice cells (the adjacent bounding cubes shown in Fig. 21). This requires some careful planning, as one unit-size lattice cell can be temporarily exceeded by the corners of a half-block during a rotation. The low density of both our meta-module and the configuration with two meta-modules in the same lattice cell (see Fig. 21), makes this possible, as illustrated in Appendix B.

Let us finally show that the robot stays connected throughout scrunch/relax, and transfer. First, notice that the sequence of moves—illustrated in Appendix B—moves each 4-unit chain as a whole, i.e., without ever disconnecting any of its units. Furthermore, every step keeps the corresponding 4-unit chain attached to the static host meta-module. In a transfer or a relax, the fact that only the guest meta-module moves, while the host meta-module(s) and all the remaining units of the robots stay still, guarantees the connectivity of the overall structure. In a scrunch, the same property holds, assuming that the meta-module to be scrunched is not a cut vertex of the graph of attachments of the robotic system. This last requirement is always satisfied in the algorithm in Butler and Rus (2003) as well as in the methods in Aloupis et al. (2011) and Vassilvitskii et al. (2002). $\square$

We remark that some reconfiguration algorithms such as Rus and Vona (2001) require each module to be able to pull or push an arbitrary number of other modules. This is a very strong requirement, and for such linear-strength algorithms we cannot avoid the use of meta-meta-modules.

It should be noted that the execution of the different operations requires many careful movements. Moreover, it requires that the units fully instantiate edge-hinged modular robots. Our algorithm comprises 51 independent movements of the six arms of the moving meta-module for the scrunch operation and 63 for the transfer operation. Refer to Appendix B for an illustration of the movements step by step. These movements can be parallelized. Indeed, 36 parallel arm movements are enough for the scrunch operation and 34 for the transfer operation, as described in Appendix C. Visualizations of all the moves are provided in a video accompanying this paper.

**Fig. 22:**
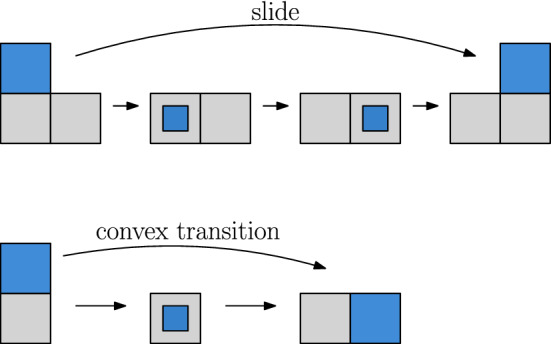
Performing slide and convex transition operations using scrunch, transfer, and relax moves

Furthermore, the fact that the edge-hinged meta-module can perform scrunch/relax and transfer operations implies that it can also perform *slide* and *convex transition* operations, illustrated in Fig. 22. This can be done by concatenating scrunch, transfer, and relax moves. Thus, the meta-module can also be used in all reconfiguration algorithms that are based on surface strategies, such as Abel and Kominers (2011), Dumitrescu and Pach (2006) and Fitch et al. (2003).

## Discussion

Meta-modules allow to greatly extend the functionality of modular robotic systems and allow them to mimic other systems. This is particularly interesting, since the design and production of units that, for instance, only rotate, can be technically simpler than that of units that can expand and contract. Therefore, the use of meta-modules provides a way to add operations such as the ability to expand and contract, which increases the reconfiguration potential of the system significantly. However, the use of meta-modules increases the number of modules needed, i.e., the grain of robot configurations as well as the synchronization requirements to implement the meta-module moves. Further enhancing the capabilities of modular robots while keeping them small is still an interesting challenge, specially in the perspective of progressive miniaturization provided by nanotechnology. Moreover, the capability increase provided by meta-modules may be interesting in order to simplify the manufacture of modular robots units while maintaining or even enhancing their versatility. For these reasons, meta-modules have been receiving attention up to this day (Aloupis et al. 2013; Dewey et al. 2008; Hurtado et al. 2015; Kawano 2020, 2019; Kotay and Rus 2000; Murata and Kurokawa 2012; Nguyen et al. 2000).

In this work we have proposed two abstract geometric classes of modular robots, edge-hinged and central-point-hinged modular robots, which cover a wide range of popular modular robots. The first one is fully instantiated by M-TRAN, Superbot, SMORES, PolyBot, and CKBot U-bar and partially instantiated by UBot and CKBot L7. The second one is instantiated by Molecubes and Roomboots. For each of these two classes we have presented a new meta-module that can simulate the expanding and contracting properties of Crystalline and Telecube robots. By presenting our meta-modules for the two proposed abstract geometric models, we ensure that their design and properties are valid will also be valid for future modular robot designs as long as they satisfy the geometric description of each model.

The proposed meta-modules represent a considerable improvement over the previously existing ones. Each meta-module is made of only 12 robot units. When expanded, the size of its bounding box is $8\times 8\times 8$, and we have proven that it is optimal. The number of units of our meta-modules is greatly reduced with respect to the 58-units meta-module presented in Aloupis et al. (2013) to less than one forth, and its size is reduced exactly to one quarter. In comparison, it is also much more compact. Furthermore, robustness is also improved over the previous meta-module, as the new meta-modules have only two corner joints per arm, as opposed to the four used in previous work. This makes operations such as alignment easier to perform. Therefore, the novel meta-modules improve over previous results in the number of units required and the space used, and have a more compact and robust structure.

Another important property of our meta-module for edge-hinged modular robots is that, in addition to being able to expand, contract, attach and detach, tunneling reconfiguration algorithms can be applied to edge-hinged modular robots without requiring the use of meta-meta-modules. We conjecture that a similar reduction can be proved for central-point-hinged modular robots. However, our proofs for the edge-hinged case cannot be applied to central-point-hinged robots due to their different characteristics.

It is also worth noticing the symmetry of the proposed meta-modules as well as the important role played by the orientation of the rotation axes of their arms in their efficient design. Last, we want to call the attention of the reader to the importance of the geometric design of units’ shapes in order to enhance feasible movements and produce a compact meta-module. For the edge-hinged units to be able to rotate, the intersection of the cubes with cylinders is essential. In the central-point-hinged case, the intersection with a cylinder is not essential to produce rotations, but it is so in order for such rotations to be feasible in the presence of neighboring units in adjacent lattice cells.

Finally, it should be noted that while the focus on this paper has been on designing a compact meta-module that provides expand/contract capabilities to a wide range of existing modular robots, there are other aspects of a meta-module that can be important and worth considering. For instance, analyzing possible strategies in case that a (meta-)module fails, such as replacement or self-healing, would be interesting from the point of view of the meta-module design and also from the algorithmic perspective.

## Conclusions

In this work we have presented two new expanding and contracting meta-modules that can be applied to a large class of modular robots that cannot expand/contract, allowing them to use tunneling strategies including efficient universal reconfiguration algorithms. Moreover, the meta-module for edge-hinged modular robots can perform scrunch/relax and transfer moves, allowing to also use surface strategies for reconfiguration, which are most common. For this case, both tunneling and surface algorithms apply without the need of meta-meta-modules.

The proposed meta-modules are optimal in size, considerably smaller, and more robust than the best previous meta-module (Aloupis et al. 2013).

## Supplementary Information

Below is the link to the electronic supplementary material.

Supplementary material 1 (pdf 6174 KB)

Supplementary material 2 (mp4 108 KB)

Supplementary material 3 (mp4 1688 KB)

Supplementary material 4 (mp4 2355 KB)

Supplementary material 5 (mp4 493 KB)

## References

[CR1] Abel, Z., & Kominers, S. D. (2011). Universal reconfiguration of (hyper-)cubic robots. ArXiv e-Prints, arXiv:0802.3414v3.

[CR2] Aloupis G, Benbernou N, Damian M, Demaine ED, Flatland R, Iacono J, Wuhrer S (2013). Efficient reconfiguration of lattice-based modular robots. Computational Geometry: Theory and Applications.

[CR3] Aloupis G, Collette S, Damian M, Demaine ED, Flatland R, Langerman S, O’Rourke J, Pinciu V, Ramaswami S, Sacristán V, Wuhrer S (2011). Efficient constant-velocity reconfiguration of crystalline robots. Robotica.

[CR4] Aloupis G, Collette S, Damian M, Demaine ED, Flatland R, Langerman S, O’Rourke J, Ramaswami S, Sacristán V, Wuhrer S (2009). Linear reconfiguration of cube-style modular robots. Computational Geometry: Theory and Applications.

[CR5] Aloupis, G., Collette, S., Demaine, E. D., Langerman, S., Sacristán, V., & Wuhrer, S. (2008). Reconfiguration of cube-style modular robots using $$O(\log n)$$ parallel moves. In *Proceedings of 19th Annual International Symposium on Algorithms and Computation (ISAAC)*, pp. 342–353.

[CR6] Brunete A, Ranganath A, Segovia S, Perez de Frutos J, Hernando M (2017). Current trends in reconfigurable modular robots design. International Journal of Advanced Robotic Systems.

[CR7] Butler Z, Rus D (2003). Distributed planning and control for modular robots with unit-compressible modules. International Journal of Advanced Robotic Systems.

[CR8] Davey, J., Kwok, N., & Yim, M. (2012). Emulating self-reconfigurable robots—design of the SMORES system. In *Proceedings of IEEE/RSJ International Conference on Intelligent Robots and Systems (IROS)*, pp. 4464–4469.

[CR9] Dewey, D. J., Ashley-Rollman, M. P., De Rosa, M., Goldstein, S. C., Mowry, T. C., Srinivasa, S. S., et al. (2008). Generalizing metamodules to simplify planning in modular robotic systems. In *Proceedings of IEEE/RSJ International Conference on Intelligent Robots and Systems (IROS)*, pp. 1338–1345.

[CR10] Dumitrescu A, Pach J (2006). Pushing squares around. Graphs and Combinatorics.

[CR11] Fitch, R., Butler, Z., & Rus, D. (2003). Reconfiguration planning for heterogeneous self-reconfiguring robots. In *Proceedings of IEEE/RSJ International Conference on Intelligent Robots and Systems (IROS)*, pp. 2460–2467.

[CR12] Hamlin G, Sanderson A (1998). Tetrobot: A modular approach to reconfigurable parallel robotics.

[CR13] Hurtado F, Molina E, Ramaswami S, Sacristán V (2015). Distributed reconfiguration of 2D lattice-based modular robotic systems. Autonomous Robots.

[CR14] Kawano, H. (2019). Linear heterogeneous reconfiguration of cubic modular robots via simultaneous tunneling and permutation. In *Proceedings of IEEE International Conference on Robotics and Automation (ICRA)*, pp. 332–338.

[CR15] Kawano H (2020). Distributed tunneling reconfiguration of cubic modular robots without meta-modules disassembling in severe space requirement. Robotics and Autonomous Systems.

[CR16] Kotay, K. D., & Rus, D. (2000). Algorithms for self-reconfiguring molecule motion planning. In *Proceedings of IEEE/RSJ International Conference on Intelligent Robots and Systems (IROS)*, pp. 2184–2193.

[CR17] Kurokawa H, Tomita K, Kamimura A, Kokaji S, Hasuo T, Murata S (2008). Distributed self-reconfiguration of M-TRAN III modular robotic system. International Journal of Robotics Research.

[CR18] Lyder, A., Garcia, R., & Stoy, K. (2008). Mechanical design of Odin, an extendable heterogeneous deformable modular robot. In *Proceedings of IEEE/RSJ International Conference on Intelligent Robots and Systems (IROS)*, pp. 883–888.

[CR19] Mounarak P, Ben-Tzvi P (2012). Modular and reconfigurable mobile robotics. Robotics and Autonomous Systems.

[CR20] Murata S, Kurokawa H (2012). Self-organizing robots.

[CR21] Nguyen, A., Guibas, L., & Yim, M. (2000). Controlled module density helps reconfiguration planning. In *Proceedings of 4th International Workshop on Algorithmic Foundations of Robotics (WAFR)*, pp. 23–25.

[CR22] Pamecha, A., Chiang, C.-J., Stein, D., & Chirikjian, G. (1996). Design and implementation of metamorphic robots. In *Proceedings of ASME Design Engineering Technical Conference & Computers in Engineering Conference (IDETC/CIE)*, pp. 18–22.

[CR23] Park, M., & Yim, M. (2009). Distributed control and communication fault tolerance for the CKBot. In *Proceedings of ASME/IFToMM International Conference on Reconfigurable Mechanisms and Robots (ReMAR)*, pp. 682–688.

[CR24] Perera, M. (2015). *Reconfiguración distribuida de robots cristalinos (Distributed reconfiguration of crystalline robots)*. Degree thesis under the supervision of V. Sacristán, Facultat d’Informàtica de Barcelona, Universitat Politècnica de Catalunya.

[CR25] Ryland, G. G., & Cheng, H. H. (2010). Design of iMobot, an intelligent reconfigurable mobile robot with novel locomotion. In *Proceedings of IEEE International Conference on Robotics and Automation (ICRA)*, pp. 60–65.

[CR26] Rus D, Vona M (2001). Crystalline robots: Self-reconfiguration with compressible unit modules. Autonomous Robots.

[CR27] Salemi, B., Moll, M., & Shen, W.-M. (2006). Superbot: A deployable, multi-functional, and modular self-reconfigurable robotic system. In *Proceedings of IEEE/RSJ International Conference on Intelligent Robots and Systems (IROS)*, pp. 3636–3641.

[CR28] Sirajoulis GC, Adamatzky A (2015). Robots and Lattice Automata.

[CR29] Spröwitz A, Pouya S, Bonardi S, van den Kieboom J, Möckel R, Billard A, Dillenbourg P, Ijspeert AJ (2010). Roombots: Reconfigurable robots for adaptive furniture. IEEE Computational Intelligence Magazine.

[CR30] Suh, J. W., Homans, S. B., & Yim, M. (2002). Telecubes: Mechanical design of a module for self-reconfigurable robotics. In *Proceedings of IEEE International Conference on Robotics and Automation (ICRA)*, pp. 4095–4101.

[CR31] Vassilvitskii, S., Yim, M., & Suh, J. (2002). A complete, local and parallel reconfiguration algorithm for cube style modular robots. In *Proceedings of IEEE International Conference on Robotics and Automation (ICRA)*, pp. 117–122.

[CR32] Yang Z, Wu Y, Fu Z, Fei J, Zheng H (2018). A unit-compressible modular robotic system and its self-configuration strategy using meta-module. Robotics and Computer Integrated Manufacturing.

[CR33] Yim, M. (1994). New locomotion gaits. In *Proceedings of IEEE International Conference on Robotics and Automation (ICRA)*, pp. 2508–2514.

[CR34] Yim M, Shen W, Salemi B, Rus D, Moll M, Lipson H (2007). Modular self-reconfigurable robot systems [grand challenges of robotics]. IEEE Robotics & Automation Magazine.

[CR35] Yim M, Zhang Y, Roufas K, Duff D, Eldershaw C (2002). Connecting and disconnecting for chain self-reconfiguration with PolyBot. IEEE/ASME Transactions on Mechatronics.

[CR36] Zhao J, Cui X, Zhu Y, Tang S (2012). UBot: A new reconfigurable modular robotic system with multimode locomotion ability. Industrial Robot.

[CR37] Zykov, V., Chan, A., Lipson, H. (2007). Molecubes: An open-source modular robotics kit. In *Workshop on self-reconfigurable robotics at IROS*.

