# Supplementary material for: A new meta-module design for efficient reconfiguration of modular robots
Source: Auton Robots. 2021 Mar 22;45(4):457–72. doi: 10.1007/s10514-021-09977-6 (PMC8549976; doi:10.1007/s10514-021-09977-6)
Supplement: Supplementary file 1 — Supplementary material 1 (pdf 6174 KB) [file 10514_2021_9977_MOESM1_ESM.pdf]

A Supplementary material: Videos

To illustrate the operations of the proposed meta-module we include videos as electronic supplementary material showing each of the operations.

A.1 Edge-hinged: Expand-contract

`edge-hinged_metamodule-expand-contract.mp4`

A.2 Edge-hinged: Scrunch

`edge-hinged_metamodule-scrunch.mp4`

A.3 Edge-hinged: Transfer

`edge-hinged_metamodule-transfer.mp4`

A.4 Central-point-hinged: Expand-contract

`central-point-hinged_metamodule-expand-contract.mp4`

B Supplementary material: Step-by-step images

In addition to the videos, the detailed step–by–step images of the scrunch and transfer operations are presented in this section.

B.1 Scrunch operation

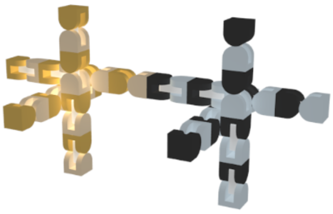

Fig. 23: Scrunch – step 0

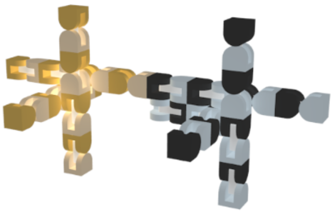

Fig. 24: Scrunch – step 1

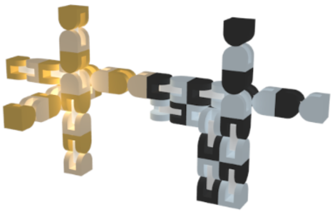

Fig. 25: Scrunch – step 2

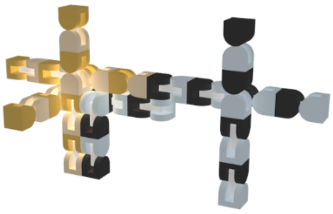

Fig. 26: Scrunch – step 3

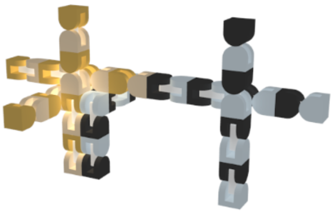

Fig. 27: Scrunch – step 4

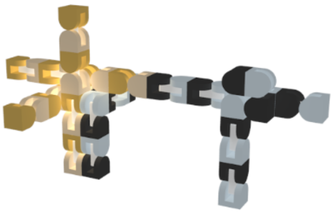

Fig. 28: Scrunch – step 5

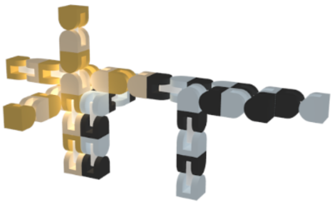

Fig. 29: Scrunch – step 6

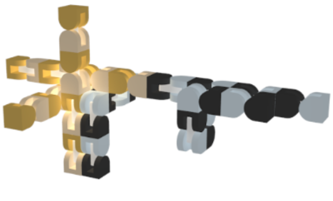

Fig. 30: Scrunch – step 7

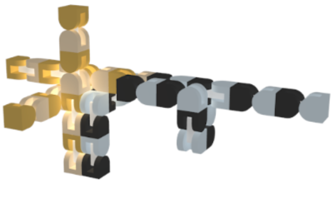

Fig. 31: Scrunch – step 8

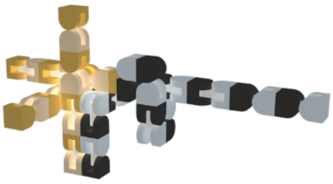

Fig. 32: Scrunch – step 9

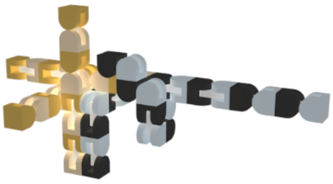

Fig. 33: Scrunch – step 10

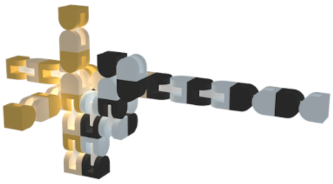

Fig. 34: Scrunch – step 11

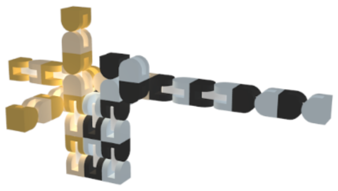

Fig. 35: Scrunch – step 12

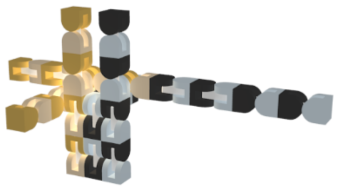

Fig. 36: Scrunch – step 13

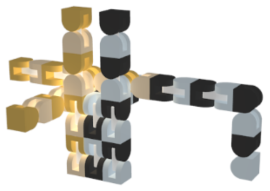

Fig. 37: Scrunch – step 14

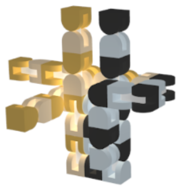

Fig. 38: Scrunch – step 15

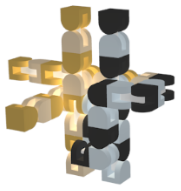

Fig. 39: Scrunch – step 16

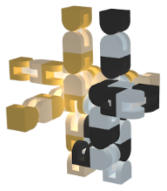

Fig. 40: Scrunch – step 17

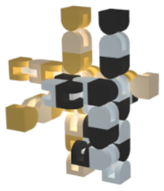

Fig. 41: Scrunch – step 18

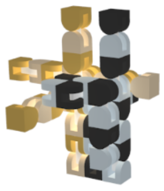

Fig. 42: Scrunch – step 19

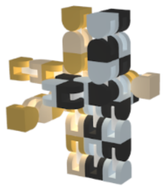

Fig. 43: Scrunch – step 20

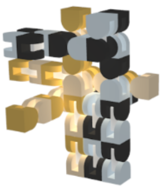

Fig. 44: Scrunch – step 21

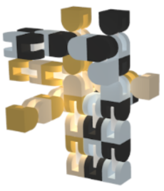

Fig. 45: Scrunch – step 22

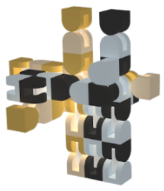

Fig. 46: Scrunch – step 23

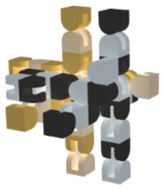

Fig. 47: Scrunch – step 24

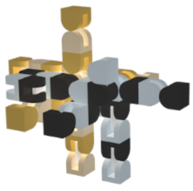

Fig. 48: Scrunch – step 25

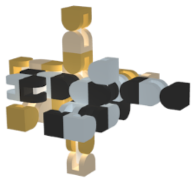

Fig. 49: Scrunch – step 26

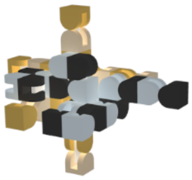
Fig. 50: Scrunch – step 27

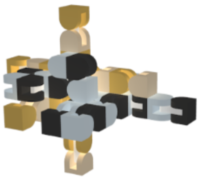
Fig. 51: Scrunch – step 28

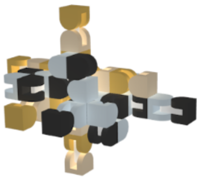
Fig. 52: Scrunch – step 29

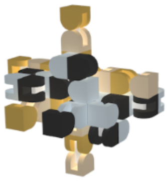
Fig. 53: Scrunch – step 30

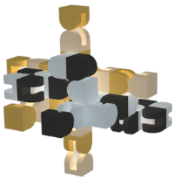
Fig. 54: Scrunch – step 31

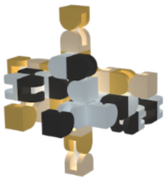
Fig. 55: Scrunch – step 32

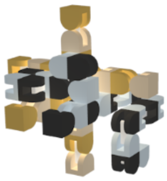
Fig. 56: Scrunch – step 33

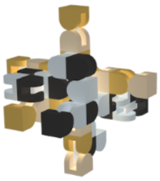
Fig. 57: Scrunch – step 34

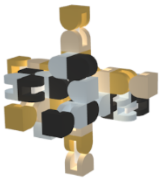
Fig. 58: Scrunch – step 35

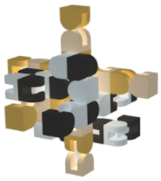
Fig. 59: Scrunch – step 36

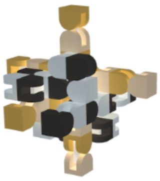
Fig. 60: Scrunch – step 37

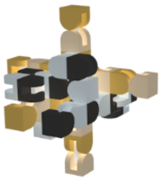
Fig. 61: Scrunch – step 38

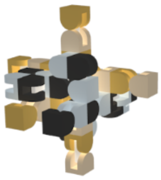
Fig. 62: Scrunch – step 39

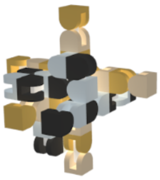
Fig. 63: Scrunch – step 40

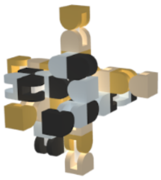
Fig. 64: Scrunch – step 41

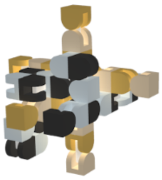

Fig. 65: Scrunch – step 42

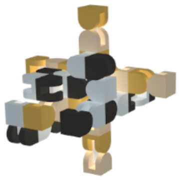

Fig. 66: Scrunch – step 43

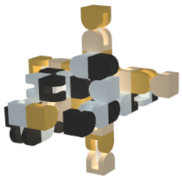

Fig. 67: Scrunch – step 44

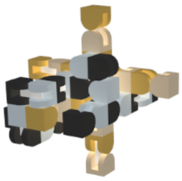

Fig. 68: Scrunch – step 45

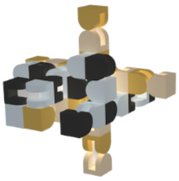

Fig. 69: Scrunch – step 46

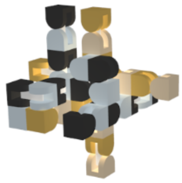

Fig. 70: Scrunch – step 47

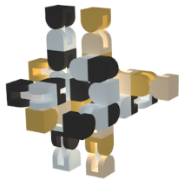

Fig. 71: Scrunch – step 48

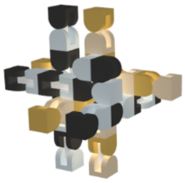

Fig. 72: Scrunch – step 49

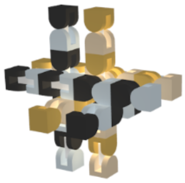

Fig. 73: Scrunch – step 50

B.2 Transfer operation

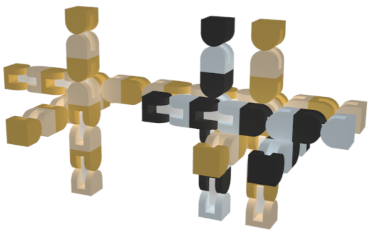

Fig. 74: Transfer – step 0

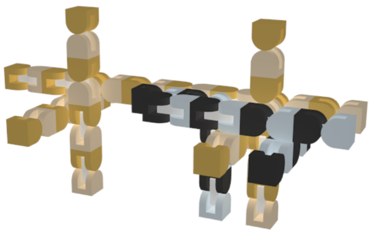

Fig. 75: Transfer – step 1

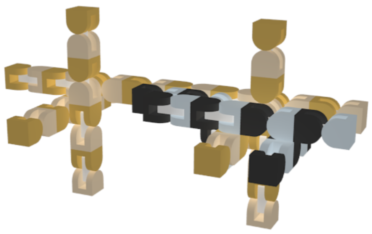

Fig. 76: Transfer – step 2

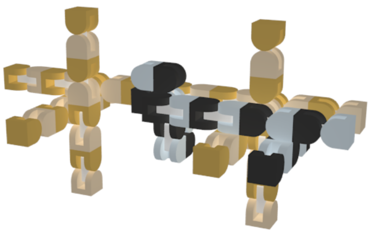

Fig. 77: Transfer – step 3

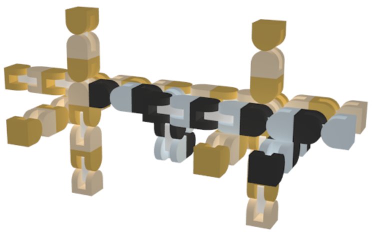

Fig. 78: Transfer – step 4

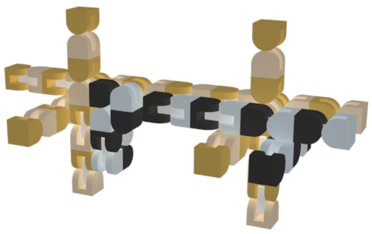

Fig. 79: Transfer – step 5

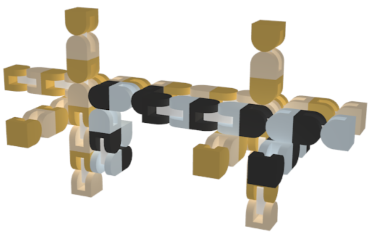

Fig. 80: Transfer – step 6

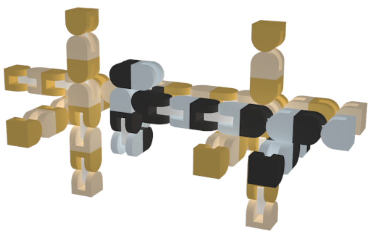

Fig. 81: Transfer – step 7

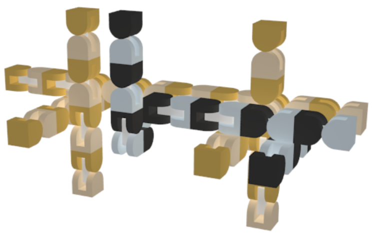

Fig. 82: Transfer – step 8

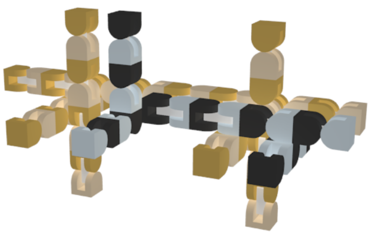

Fig. 83: Transfer – step 9

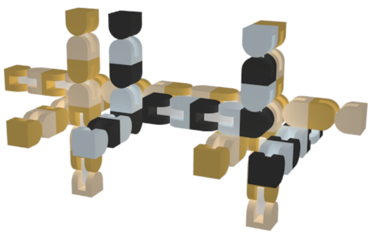

Fig. 84: Transfer – step 10

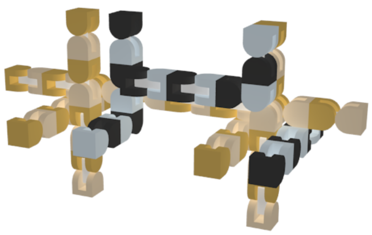

Fig. 85: Transfer – step 11

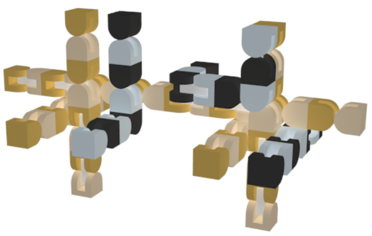

Fig. 86: Transfer – step 12

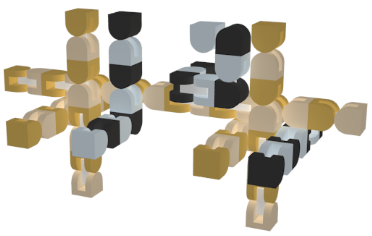

Fig. 87: Transfer – step 13

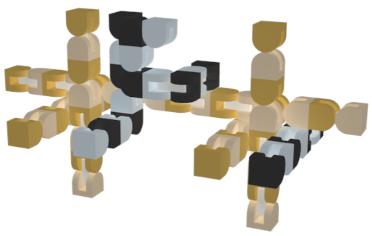

Fig. 88: Transfer – step 14

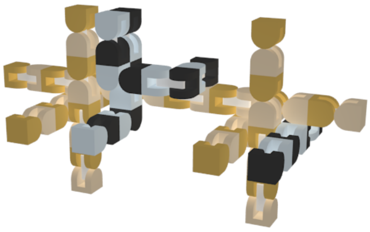
Fig. 89: Transfer – step 15

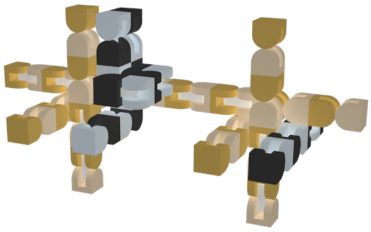
Fig. 90: Transfer – step 16

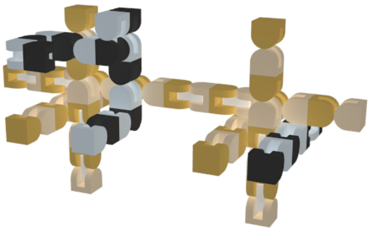
Fig. 91: Transfer – step 17

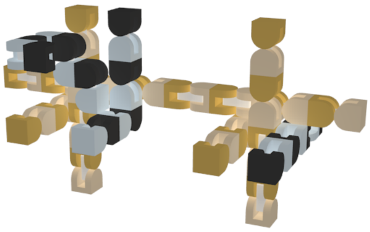
Fig. 92: Transfer – step 18

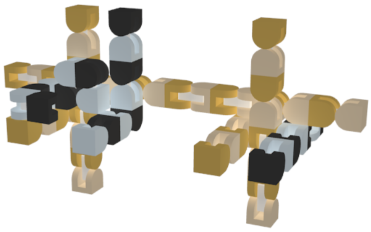
Fig. 93: Transfer – step 19

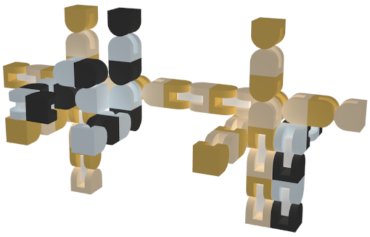
Fig. 94: Transfer – step 20

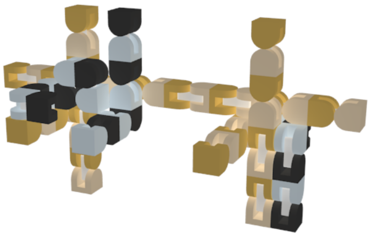
Fig. 95: Transfer – step 21

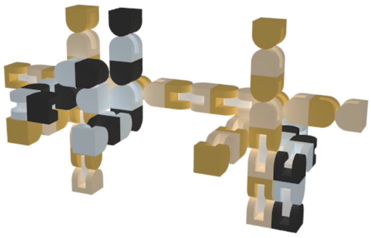
Fig. 96: Transfer – step 22

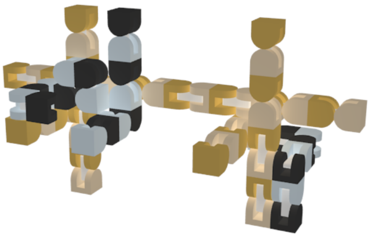
Fig. 97: Transfer – step 23

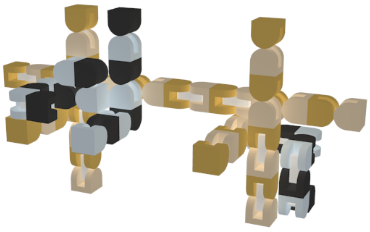
Fig. 98: Transfer – step 24

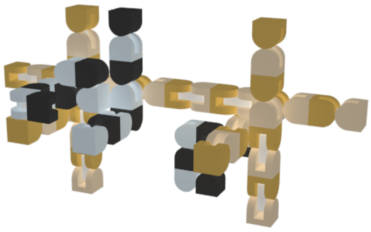
Fig. 99: Transfer – step 25

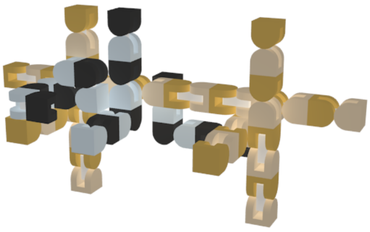
Fig. 100: Transfer – step 26

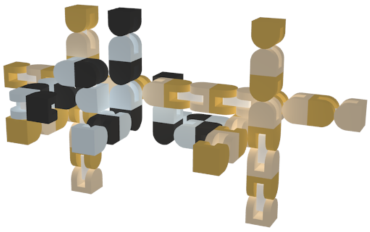
Fig. 101: Transfer – step 27

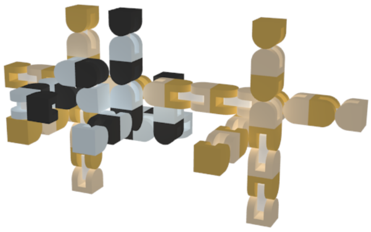
Fig. 102: Transfer – step 28

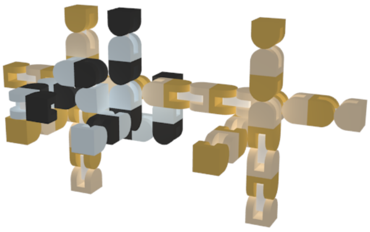
Fig. 103: Transfer – step 29

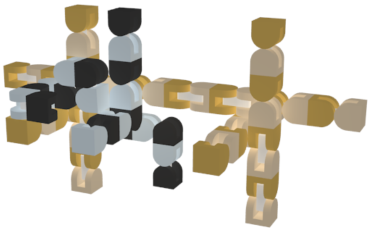

Fig. 104: Transfer – step 30

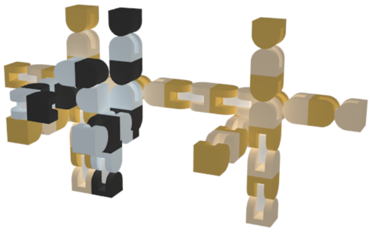

Fig. 105: Transfer – step 31

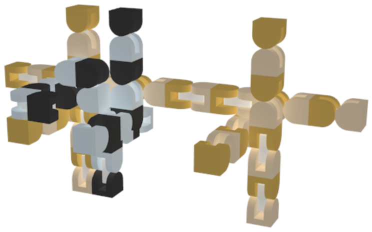

Fig. 106: Transfer – step 32

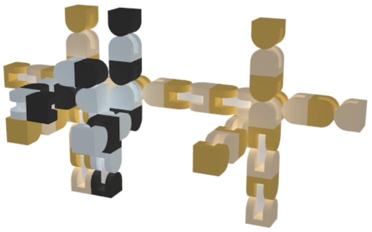

Fig. 107: Transfer – step 33

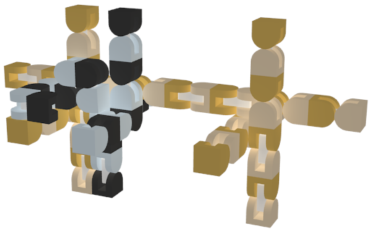

Fig. 108: Transfer – step 34

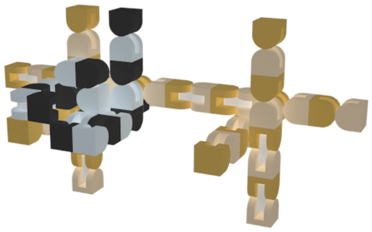

Fig. 109: Transfer – step 35

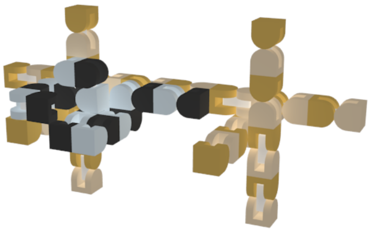

Fig. 110: Transfer – step 36

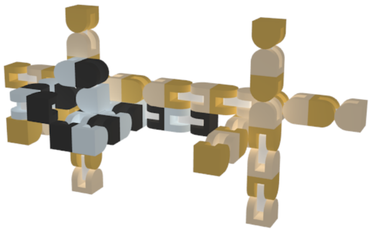

Fig. 111: Transfer – step 37

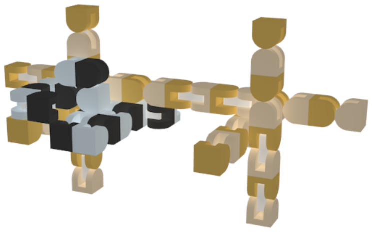

Fig. 112: Transfer – step 38

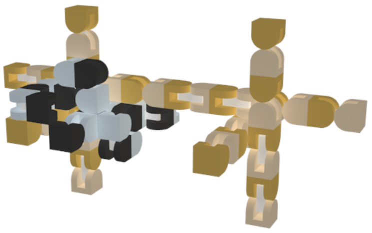

Fig. 113: Transfer – step 39

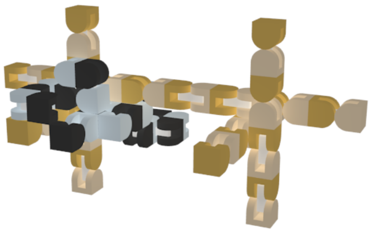

Fig. 114: Transfer – step 40

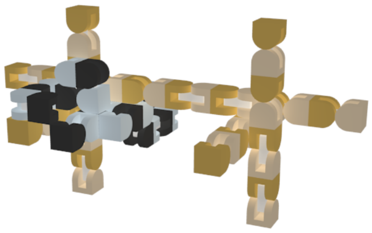

Fig. 115: Transfer – step 41

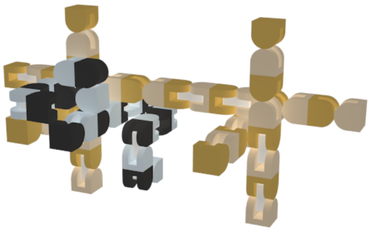

Fig. 116: Transfer – step 42

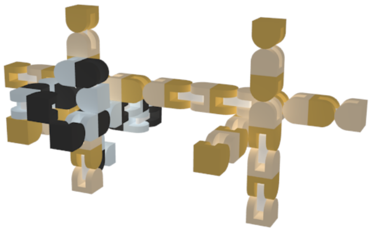

Fig. 117: Transfer – step 43

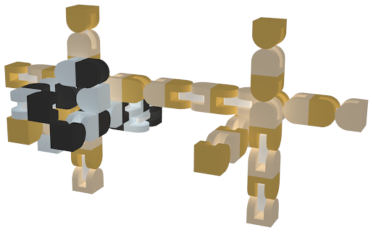

Fig. 118: Transfer – step 44

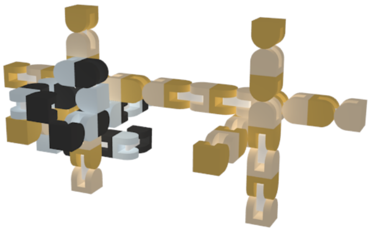

Fig. 119: Transfer – step 45

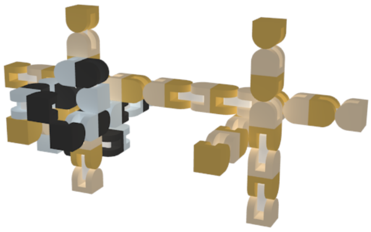

Fig. 120: Transfer – step 46

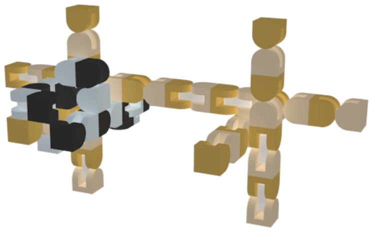

Fig. 121: Transfer – step 47

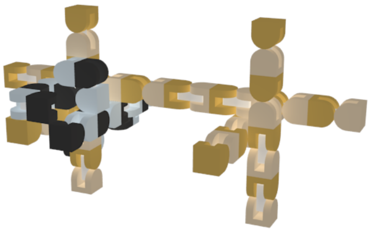

Fig. 122: Transfer – step 48

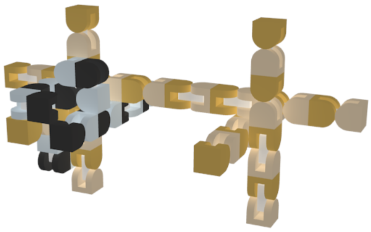

Fig. 123: Transfer – step 49

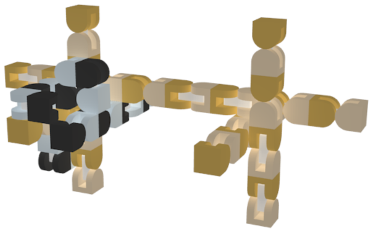

Fig. 124: Transfer – step 50

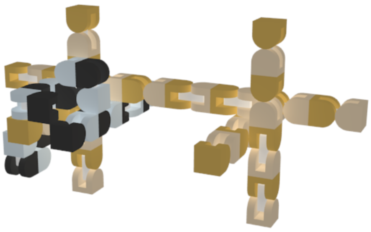

Fig. 125: Transfer – step 51

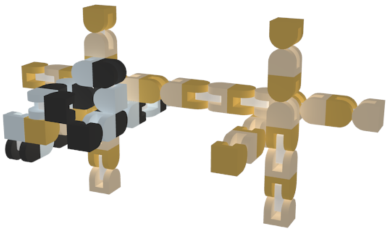

Fig. 126: Transfer – step 52

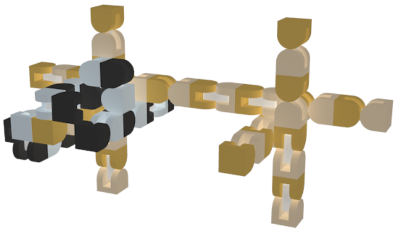

Fig. 127: Transfer – step 53

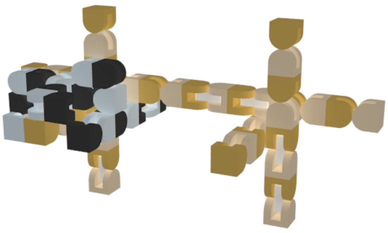

Fig. 128: Transfer – step 54

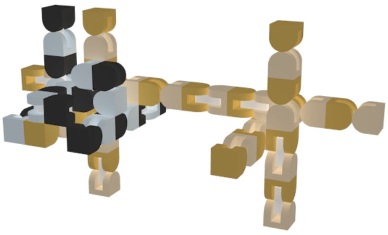

Fig. 129: Transfer – step 55

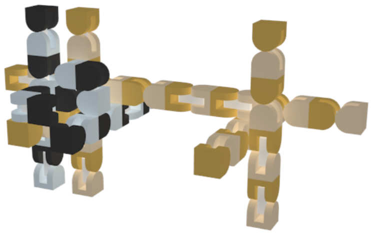

Fig. 130: Transfer – step 56

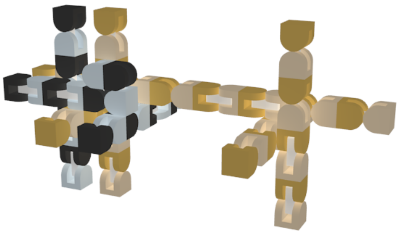

Fig. 131: Transfer – step 57

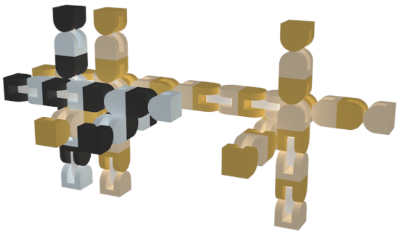

Fig. 132: Transfer – step 58

C Supplementary material: Parallelization

In this section we present tables showing how the steps for the scrunch and transfer operations can be parallelized.

Parallel steps	1	2	3	4	5	6	7	8	9	10	11	12	13	14	15	16	17	18
Scrunch steps<br>(as in Appendix B)	1<br>2	3<br>5	4<br>6<br>7	8	9<br>14	10<br>16	11	12<br>15	13<br>17	18	19<br>20	21<br>24	22	23	25<br>45	26	27	28

Parallel steps	19	20	21	22	23	24	25	26	27	28	29	30	31	32	33	34	35	36
Scrunch steps<br>(as in Appendix B)	29	30	31	32	33	34	35	36	37	38	39	40	41	42	43	44	46	47<br>48<br>49<br>50

Table 1: Scrunch with parallel steps

Parallel steps	1	2	3	4	5	6	7	8	9	10	11	12	13	14	15	16	17	18
Transfer steps<br>(as in Appendix B)	1<br>2<br>20<br>21	3<br>22	4<br>23	5<br>24	6<br>25	7<br>26	8<br>9<br>27	12<br>28	13<br>29	14<br>30	15<br>31	16<br>32	17<br>33	18<br>34	19<br>35	36	37	38<br>39

Parallel steps	19	20	21	22	23	24	25	26	27	28	29	30	31	32	33	34
Transfer steps<br>(as in Appendix B)	40	41	42	43	44	45	46	47	48	49	50	51	52	53	54	55<br>56<br>57<br>58

Table 2: Transfer with parallel steps
